# Striving for Uniformity: A Review on Advances and Challenges To Achieve Uniform Polyethylene Glycol

**DOI:** 10.1021/acs.oprd.3c00428

**Published:** 2024-04-01

**Authors:** Cláudia Bento, Marianna Katz, Maria M. M. Santos, Carlos A. M. Afonso

**Affiliations:** † Hovione Farmaciência S.A., Estrada do Paço do Lumiar, Campus do Lumiar, Edifício R, 1649-038 Lisboa, Portugal; ‡ Research Institute for Medicines (iMed.ULisboa), Faculty of Pharmacy, Universidade de Lisboa, Avenida Professor Gama Pinto, 1649-003 Lisboa, Portugal

**Keywords:** polyethylene glycol, poly(ethylene oxide), controlled synthesis, monodisperse PEG, uniform PEG

## Abstract

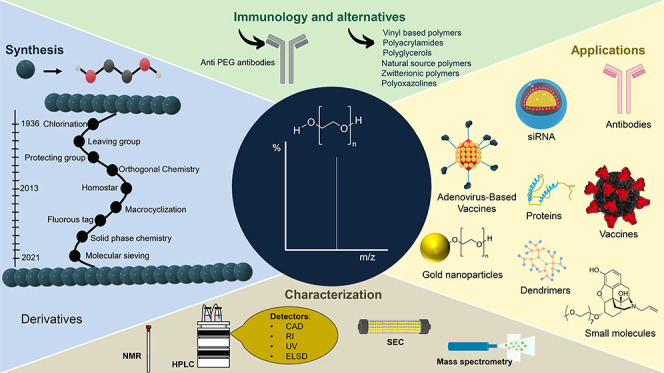

Poly(ethylene glycol) (PEG) is the polymer of choice in drug delivery systems due to its biocompatibility and hydrophilicity. For over 20 years, this polymer has been widely used in the drug delivery of small drugs, proteins, oligonucleotides, and liposomes, improving the stability and pharmacokinetics of many drugs. However, despite the extensive clinical experience with PEG, concerns have emerged related to its use. These include hypersensitivity, purity, and nonbiodegradability. Moreover, conventional PEG is a mixture of polymers that can complicate drug synthesis and purification leading to unwanted immunogenic reactions. Studies have shown that uniform PEGylated drugs may be more effective than conventional PEGylated drugs as they can overcome issues related to molecular heterogeneity and immunogenicity. This has led to significant research efforts to develop synthetic procedures to produce uniform PEGs (monodisperse PEGs). As a result, iterative step-by-step controlled synthesis methods have been created over time and have shown promising results. Nonetheless, these procedures have presented numerous challenges due to their iterative nature and the requirement for multiple purification steps, resulting in increased costs and time consumption. Despite these challenges, the synthetic procedures went through several improvements. This review summarizes and discusses recent advances in the synthesis of uniform PEGs and its derivatives with a focus on overall yields, scalability, and purity of the polymers. Additionally, the available characterization methods for assessing polymer monodispersity are discussed as well as uniform PEG applications, side effects, and possible alternative polymers that can overcome the drawbacks.

## 1 Introduction

Poly(ethylene glycol) (PEG) is a synthetic, hydrophilic, and biocompatible polymer with the chemical formula H(OCH_2_CH_2_)_*n*_OH, where *n* corresponds to the number of units of ethylene oxide. This polymer can have liquid form when molecular weights are below 1000 Da, and it is a waxy solid when molecular weights are between 1000 and 2000 Da. When the molecular weight is above 2000 Da, PEGs are hard crystalline solids. PEGs are widely used in the cosmetic, pesticide, and food processing industries, as a separation and purification aid, as matrices for embedding, as antifreezes, as lubricants for medical devices, as solvents in chemical reactions, and in suppositories and tableting as well as being used in drug delivery. In fact, the Food and Drug Administration (FDA) has selected PEG as the preferred choice for drug delivery systems because of its nontoxicity, low immunogenicity, and well-established safety profiles compared to other polymers, which are key requisites for any component used in formulation development, such as drug carriers, coatings, or excipients. The PEG chains can be covalently or noncovalently attached to small molecule drugs, peptides, and nucleic acids by a strategy called PEGylation.^1^ This technique has been shown to optimize the pharmacokinetics and pharmacodynamics of drugs, improving drug stability, and reducing nonspecific protein absorption and macrophage uptake, therefore significantly prolonging the circulation time due to the stealth effect of PEG.^2−5^ To this purpose, PEG has been chemically modified by introducing a variety of functional groups to synthesize tailored PEG derivatives, making this polymer even more suitable for clinical drug development.^6,7^ To date, over 30 PEGylated drugs have been approved for clinical applications with a market size of over USD 10 billion.^8,9^ Nevertheless, the use of PEGs has some drawbacks. In particular, increasing PEG molecular weight and hydrodynamic sizes will reduce renal filtration, enabling a long-term action of drugs and thus a significant reduction in dosing frequency. However, employing PEG with low molecular weights was found to be toxic.^10,11^ Moreover, several PEGylated therapeutics induce the development of anti-PEG antibodies (APA) in patients triggering enhanced blood clearance (ABC), causing a reduced efficacy of the products. This drawback has been associated with multiple treatments with PEGylated drugs and consumption of products containing PEG, leading to reduced efficacy and increased adverse events.^12,13^

Another downside is the conventional process that produces PEG. Due to the randomness of the process, the products are mixtures of many different molecules of varying length and molecular weight with a disperse nature.^14−17^ This overcome the hurdles the synthetic and purification process of PEGylated drugs, thereby compromising the reproductive quality. Although these mixtures are currently used for PEGylation of pharmaceuticals, they suffer from several limitations. It is hard to maintain consistent product composition across batches, the characterization is challenging, the pharmaceutical ingredient activity can be lost due to PEG size heterogeneity, and there are hurdles in obtaining FDA approval.^17−19^ To address these issues, significant efforts have been made to synthesize uniform PEGs via stepwise organic synthesis with a narrow molecular weight and a polydispersity index (PDI) of 1.0. However, these new methods usually involve step-by-step iterations which have some drawbacks such as low reaction rates and the need for chromatographic purification after every step.^20^ In this review, we summarize the synthetic efforts made in the past decades to synthesize uniform PEGs and the current limitations and challenges of PEGylation using conventional disperse PEGs, highlighting the astonishing changes of improved therapeutic efficacy and reduced cytotoxicity when disperse PEG is substituted by uniform PEG, optimizing the therapeutic outcomes and easing side effects.

## 2 Polyethylene Glycol (PEG) Synthesis

### 2.1 Disperse PEGs

Disperse PEG refers to a mixture of PEG molecules with a wide range of molecular weights or chain lengths. Usually, the PDI associated with these types of polymers has values above 1.1, and its synthesis relies mainly on ring-opening polymerization. This technique has been used since the 1930s, and it remains to be the main technique used for the PEO (poly(ethylene oxide)) produced, despite all of its limitations.^21^

**Scheme 1:**
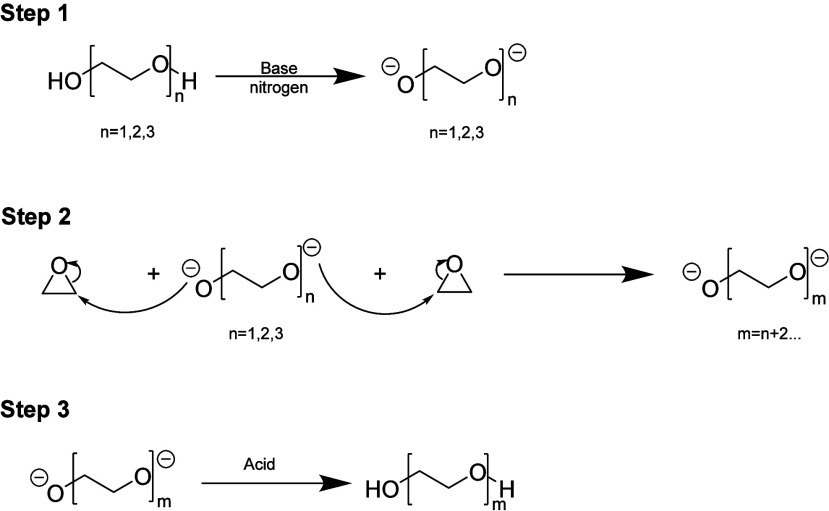
General Chemical Process To Synthesize PEG with Ethylene Oxide

Scheme 1 provides a straightforward example of how anionic ring-opening polymerization works to synthesize PEG. The first step comprises the reaction of a PEG initiator, usually a monomeric PEG molecule that is activated with a catalyst, which is typically potassium hydroxide. This PEG initiator is activated, generating the alkoxide ion. The second step comprises the reaction of the activated PEG with ethylene oxide (EO), where there is a nucleophilic attack of the alkoxide ion on the epoxide ring, resulting in the formation of a new ether bond. This process is repeated multiple times to form the polymer chain. The third step terminates the polymerization process, where a quenching agent is added, such as acetic acid, to deactivate the catalyst and prevent further polymerization.^22−24^

The molecular weight of the PEG polymer chain is determined by the number of EO monomer units that has been added to the PEG initiator. By controlling the ratio of EO monomer to PEG initiator, it is possible to tailor the molecular weight and properties of the resulting PEG polymer for different applications.^25^

Although this process is still widely used, it has several disadvantages such as polydispersity. By using this approach, several defects can happen: the reaction can be incomplete or the polymer can undergo chain-transfer reactions with other molecules or impurities resulting in a mixture or shorter or incomplete polymer chains.^26^ In an attempt to avoid these challenges, when synthesizing PEG, the reaction conditions must be completely anhydrous to ensure the purity of the product. If water is present then it will open ethylene oxide, producing PEG diols as side products.^27^

Through the years, some attempts have been done to improve this type of polymerization. To avoid the diol content, Ma et al. developed a new initiating system with potassium naphthalene and methanol being able to effectively remove the trace amount of water and oxygen in the reaction system and obtained a final product with a PDI of 1.07.^28^ To avoid the organometallic reagents used to form reactive potassium alkoxides, Bento et al. established a synthetic route that formed the propagating alkoxides by azeotropic distillation, removing water from the alcohol/alkoxide equilibrium, since the water drives the equilibrium to the potassium alkoxide without the use of organometallics. However, the PDI associated with this method is still between 1.1 and 1.4.^29^ Moreover, continuous flow chemistry was applied to this type of anionic ring polymerization of ethylene oxide and showed narrower distributions (PDI 1.06) by using MeONa as the catalyst.^30^

On the other hand, Naumann et al. performed the first successful copolymerization of PO and EO via NHO organo- and dual-catalysis conditions. Especially, the dual, Mg(II)-assisted approach (system B) represents a viable strategy to synthesize high molar mass P(PO-coEO) copolymers with molecular weights > 50 000 g mol^–1^,^31^ while Pelegri-O’Day et al. developed a ring-opening metathesis polymerization (ROMP) to synthesize unsaturated protein-reactive PEG with aldehyde functionality. To do so, polymerizations were carried out in a glovebox under a nitrogen atmosphere at 20–23 °C with ratios of [Grubbs I]:[monomer 1] equal to [1]:[10–100] and terminated by the addition of excess vinylene carbonate. Any residual active ruthenium catalyst was subsequently quenched with ethyl vinyl ether. The crude polymers were purified by precipitation into cold diethyl ether, and ruthenium could be completely removed after treatment with tris(hydroxymethyl)phosphine. However, the PDI values were quite high (between 1.13 and 1.6).^32^

Nevertheless, despite all of these achievements, ring-opening polymerization still provides products with a mixture of different molecular weights. Although these mixtures are currently used for PEGylation of pharmaceuticals, they are not ideal since they cannot achieve consistent compositions between batches. The characterization of pharmaceutical products is a challenging task, particularly due to the heterogeneity of their physical properties caused by the different sizes of PEG. As a result, there is a risk of loss of biological activities of the pharmaceutical ingredients. Moreover, obtaining FDA approval can pose a challenge due to this complexity in defining the products accurately.^19,33,34^

### 2.2 Uniform PEGs

**Figure 1:**
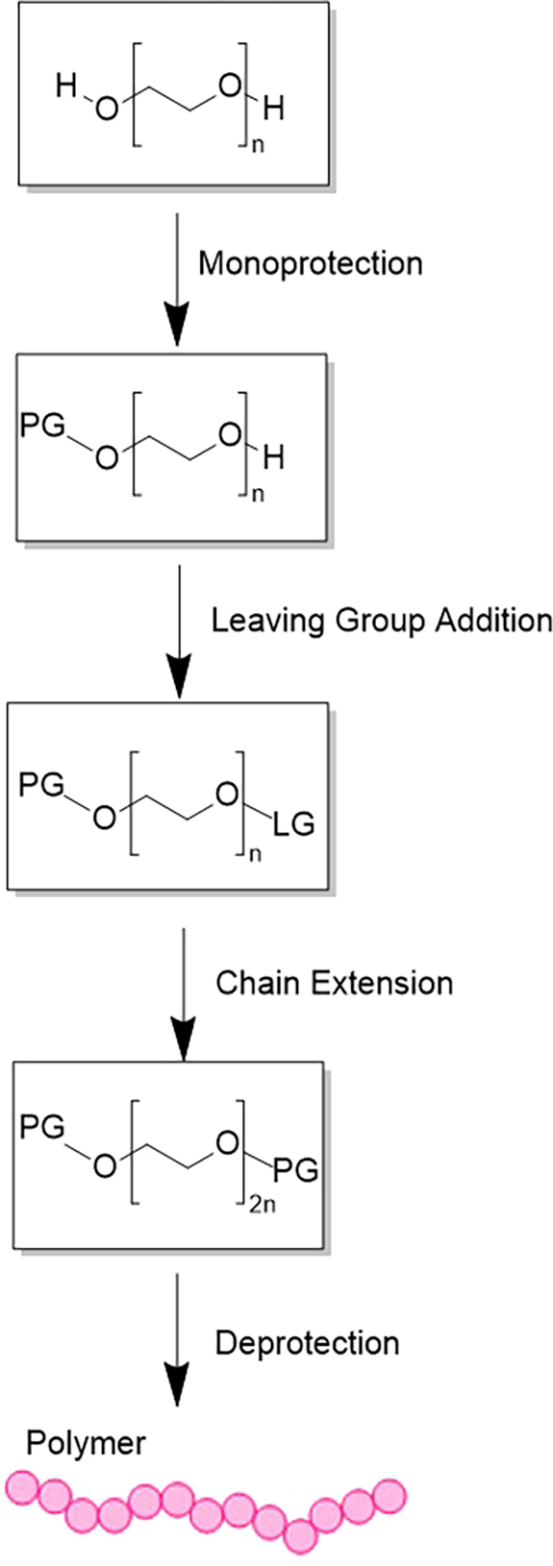
Conventional monodisperse PEG synthesis: PG, protecting group; LG, leaving group.

**Figure 2:**
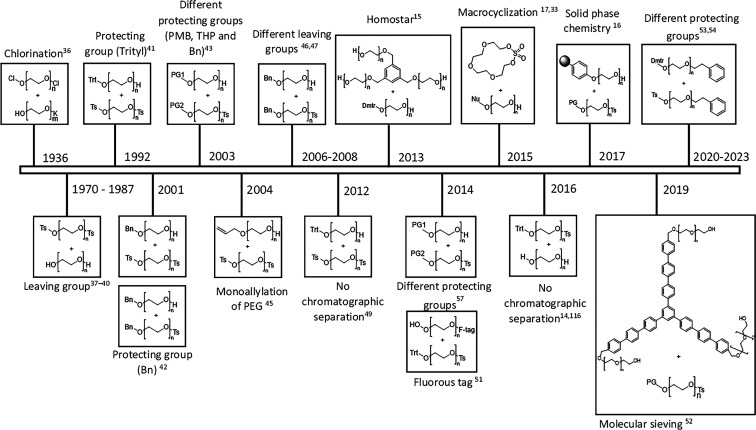
Overview of developed approaches on monodisperse PEG synthesis.

There are several different routes for stepwise uniform PEG synthesis, which include unidirectional iterative coupling, bidirectional iterative coupling, chain doubling, and chain tripling.^35^ Additionally, since alcohols make poor leaving groups, the majority of the investigated strategies include using leaving groups to encourage the chain extension reaction and the iterative application of a protective group to control the chain extension reaction (Figure 1). As a result, during the last few decades, several research groups have developed unique methods for creating uniform PEGs, as shown in Figure 2.

The attempt to synthesize uniform PEGs dates to 1936 where Fordyce first reported PEG synthesis by reacting dichloroethane with ethylene glycol alkoxides. Even though he was able to elongate the PEG chain, a mixture of different length PEGs was obtained.^36^ In 1970, Bomer et al. successfully synthesized PEG lengths of 9, 15, 27, and 45 with lower degrees of polymerization (DP). They were able to reduce the DP from 45 to 27 by using a leaving group approach with tosyl chloride and separated the oligomers by distillation; however, they still lack control over the uniformity of the chains formed.^37^ Later, in 1980, a tosylation approach was followed. However, they discovered that using sodium methoxide as the base allowed competitive reactions of sodium methoxide and the sodium salt of triethylene glycol with triethylene glycol ditosylate, which led to the formation of the dimethyl ether of triethylene glycol and the methyl ether of hexaethylene glycol.^38^ In 1982, they improved their previous work by attempting to purify the mixture obtained before by preparative gel filtration using a Sephadex LH-20 packing improving to a purity of 99%. However, it is clear that this process lacks chain control steps since the product was a mixture of PEGs with *n* = 5, 10, 15, 20, and 25.^39^ Later, in 1987, the same tosylated approach for chain extension was studied, optimizing the reported yields by employing a different base for the tosylation of the PEG step. The bases used were TEA, pyridine, DBU, and NaOH.^40^ In 1992, the reach of a PEG with 54 ethylene oxide units using this type of synthesis with tosyl chloride was first reported. They added the addition step of a monoprotecting PEG chain with a trityl group to improve the control in the reaction and avoid side reactions.^41^

**Scheme 2:**
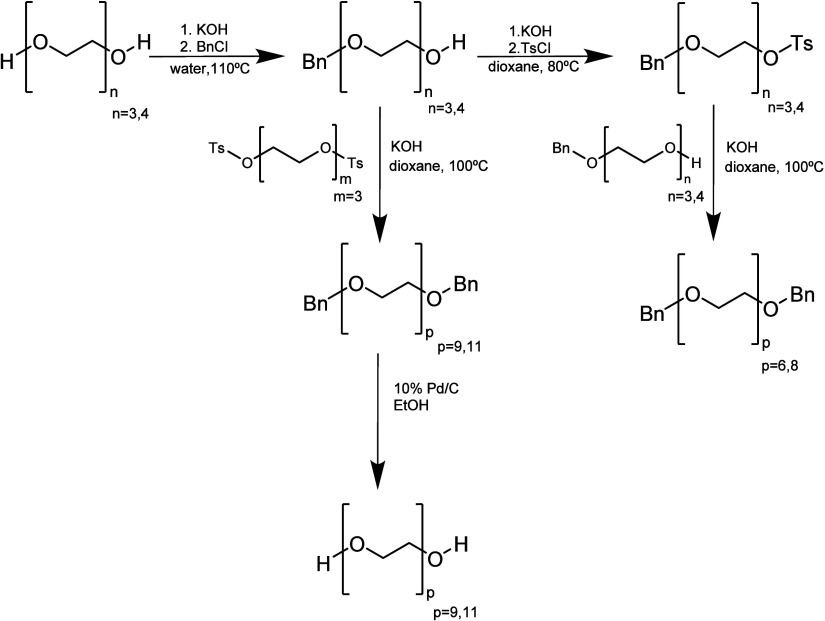
Synthetic Pathway of Uniform PEG Developed by Zada et al.^42^

In 2001, Zada et al. first reported the use of benzyl to monoprotect PEG (Scheme 2). They observed that blocking one end group of PEG with a protecting group, such as benzyl, which can be removed at a later stage in the synthesis, will not lead to the formation of a large mixture of oligomers. Moreover, they concluded that monoprotection with benzyl as the protecting group and NaH as the base improved the yields and that using more sophisticated separation and purification methods, such as preparative size-exclusion chromatography, improved the final purity.^42^

**Scheme 3:**
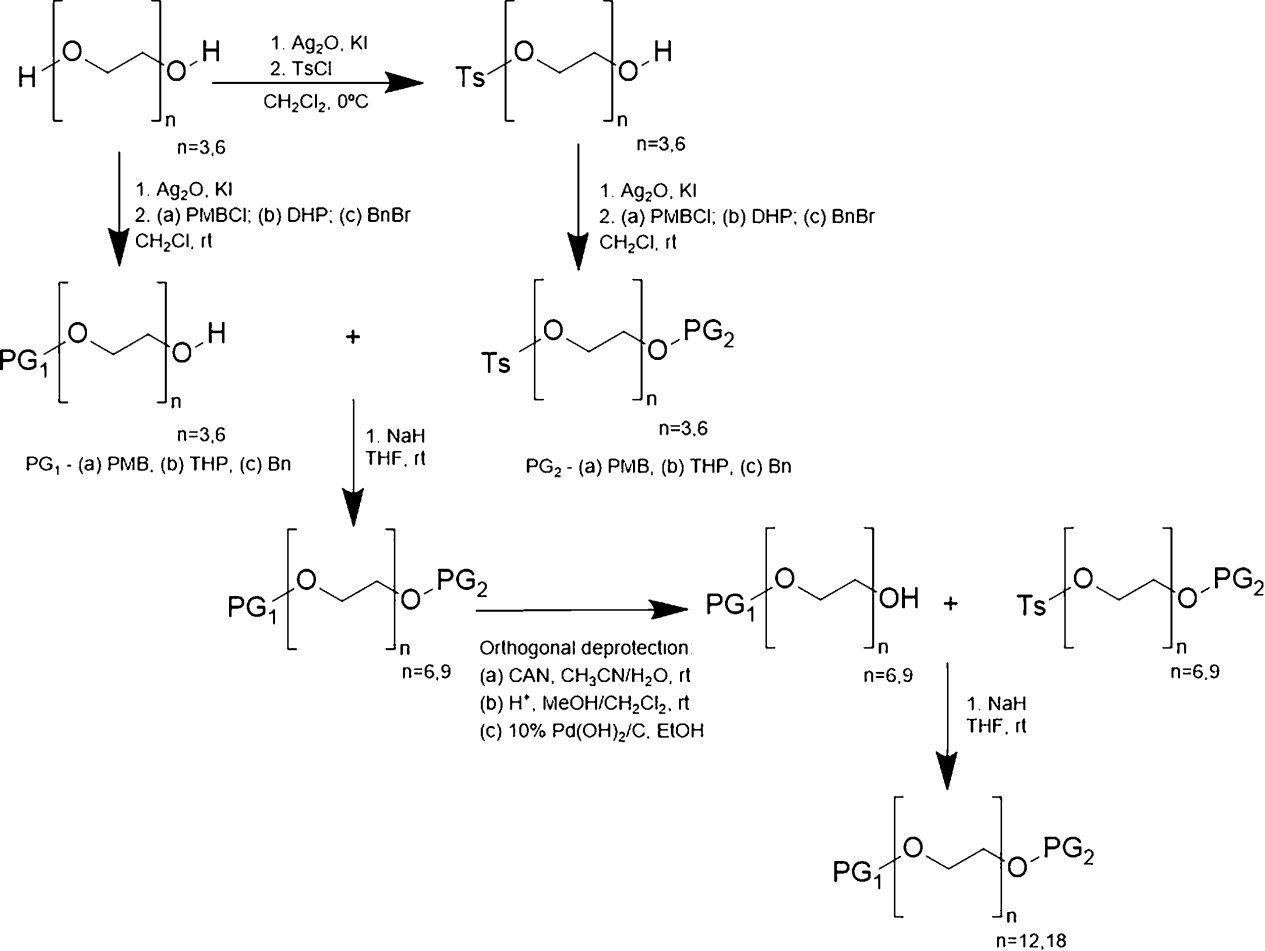
Orthogonal Protecting Group Approach for the Synthesis of Uniform PEGs

By 2003, a new approach was developed to produce elongated uniform PEGs with high yield by selectively adding asymmetric PEGs via iterative growth using an orthogonal protecting group (Scheme 3). This technique avoided the need to deprotect and protect a PEG chain in each iteration as well as the need for an excess of one of the reactants to maximize the yield of these unsymmetrical PEGs. Therefore, the protecting groups were selectively removed, providing a hydroxyl site for further chain extension. Three protecting groups were employed in this study (PMB, THP, and Bn) with different stabilities. For example, the benzylic protecting groups were stable under the acidic conditions required for the deprotection of the THP group but not vice versa. Likewise, PMB was oxidatively cleaved in the presence of Bn in high yields. Therefore, these trends impose that the selective monodeprotection of these bifunctional molecules has to be performed in the following order: THP > PMB > Bn. Although effective, this approach added one extra step to the general iterative process.^43^ A similar approach was developed in 2014 with benzyl and THP.^44^ Furthermore, different approaches arose to protect PEG in the chain extension reaction: monoallylate PEG oligomers. However, the results of this work gave poor yields (<20%).^45^ Reaching 2006, Ahmed et al. studied different leaving groups, such as tosyl, mesyl, or chloride. They found that doing the reaction with monobenzylated PEG tosylate with oligoethylene glycol provides better yields using monoprotected PEG with ditosylated PEG oligomer.^46^ Others followed this approach, such as Niculescu-Duvaz et al.,^47^ French et al.,^35^ and Maranski et al., with similar outputs.^48^

**Scheme 4:**
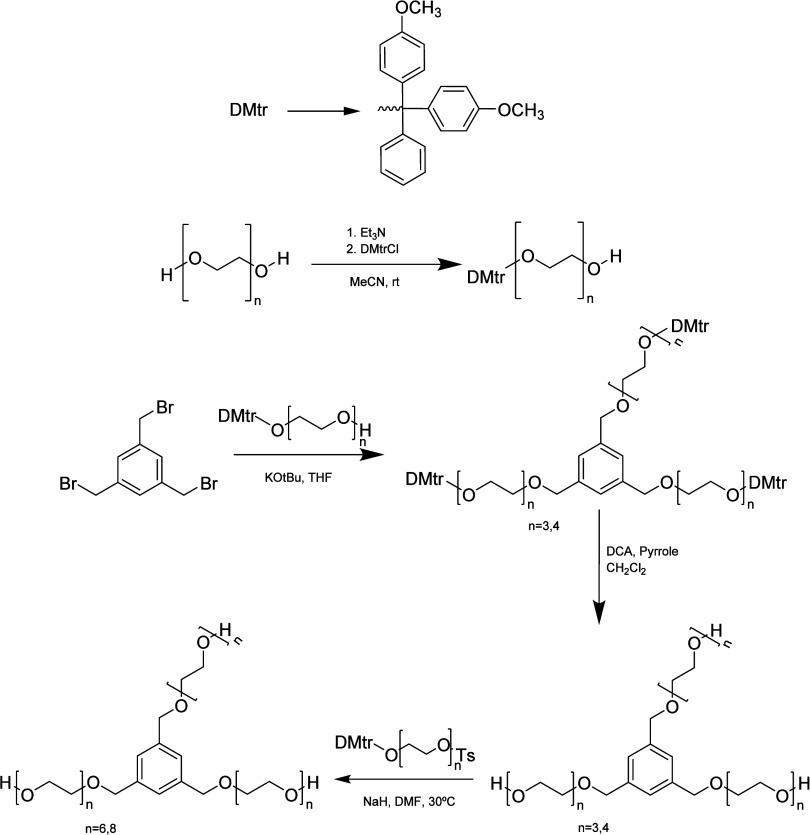
Synthesis of Uniform PEG Using a Homostar Approach

In 2011, Gothard et al. developed a synthetic approach to synthesize PEG 6, 10, and 12 without the need of column chromatographic separation. To do so, bidirectional growth of the PEG chains was made using easy-to-remove trityl groups that are easily removed in liquid–liquid extraction.^49^ In 2014, a novel homostar approach was exploited by using a hub derived from 1,3,5-tris(bromomethyl)benzene that was linked to each PEG chain through a benzyl ether that works as a protecting group (Scheme 4). The chain grown unidirectionally by attaching building blocks into each end of existing chain and the branched structure facilitated the chromatographic purification of oligomeric intermediates. Moreover, even though with this method chromatography was used to isolate products, it was noted that the large size and higher polarity of the homostar provided potential for purification by alternative size-discriminating techniques such as organic solvent nanofiltration.^15^ This homostar approach can also be used to prepare a range of heterobifunctional, uniform PEGs having useful cross-linking functionalities (−OH, −COOH, −NH_2_, −N_3_) at both ends.^50^

Later, in 2014, fluorous solid-phase extraction (FSPE) and solid-phase extraction (SPE) were employed in the synthesis of uniform PEGs. The fluorous tag was used as a protective group for the hydroxyl group in PEGs and as a fluorous separative tag for fluorous purification. During the deprotecting and coupling cycle, FSPE and normal-phase silica gel were used to efficiently purify the intermediates.^51^

**Scheme 5:**
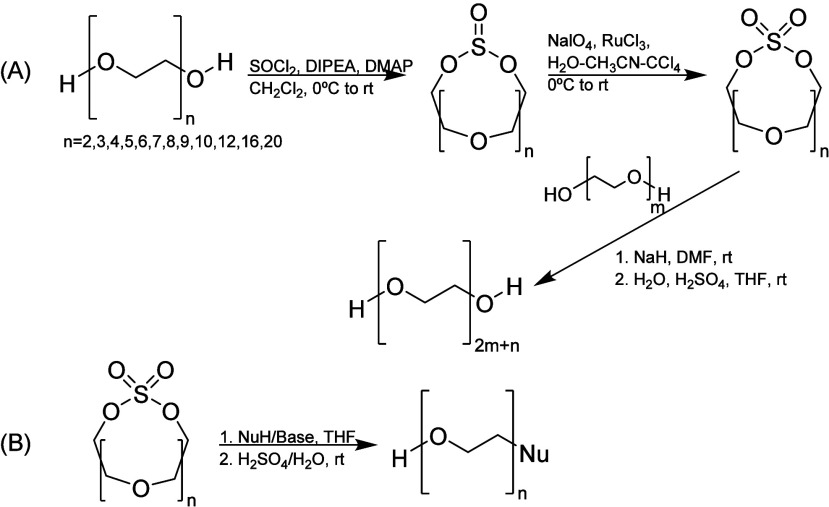
Synthetic Pathway Using a Macrocyclic Approach (A) Macrocyclization of PEGs and consecutive longer PEG synthesis. (B) Monofunctionalization of PEGs.

In 2015, a novel approach for the synthesis of monodisperse PEGs and their monofunctionalized derivatives using a macrocyclic-sulfate-based strategy was developed (Scheme 5). The macrocyclization was made with SOCl_2_ and uniform PEG, leading to macrocyclic sulfites that are further oxidized to macrocyclic sulfates. These are then applied as precursors for monofunctionalized PEGs through nucleophilic ring-opening reactions, minimizing bisfunctionalized side products. Furthermore, these macrocyclic compounds can also be used to allow a more efficient chain extension procedure with two PEG fragments, therefore avoiding the iterative protecting and leaving group approach of the −OH terminal PEG, minimizing the synthetic steps. The nucleophilic attack allowed the ease of monofunctionalization of PEG, leading to the largest uniform monomethoxy-PEGs synthesized with 64 EO units, and hydroxyl-PEG was synthesized using this approach until PEG36. However, as a disadvantage, all of the intermediates were purified by flash chromatography.^17,33^

**Scheme 6:**
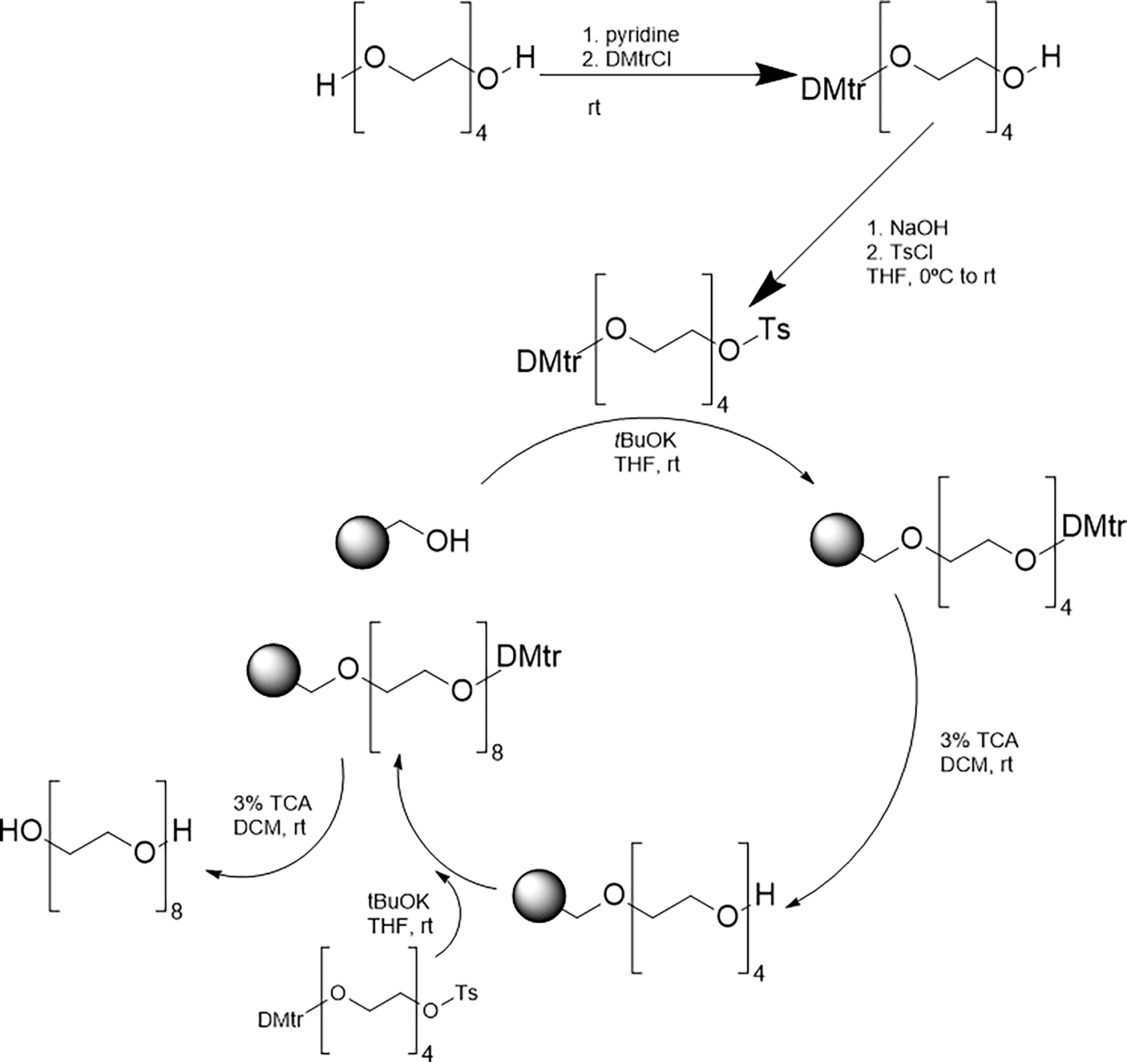
Solid-Phase Approach To Synthesize Uniform PEGs Developed by Khanal et al.

Solid-phase technology was also applied for the synthesis of uniform PEGs to attempt to avoid chromatographic separations, and Khanal et al. attempted to overcome this issue (Scheme 6). By employing solid-phase chemistry to do the uniform PEG synthesis, they were able to avoid the tedious purifications required for this procedure. The purification of intermediates and product was made by washing, and the used excess of reactants allowed high conversions, overcoming the low efficiency of the Williamson ether reaction. The pure products were obtained without chromatography until PEG12 length. However, when they attempted to move forward to higher PEG lengths, they found many challenges as they went through the synthesis of PEG16 and PEG20: some chains did not react further; therefore, it became difficult to remove shorter PEGs from longer PEGs.^16^ Later, in 2019, a novel strategy was developed combining liquid-phase synthesis and selective molecular sieving. For this purpose, a star-shaped macromolecule was used that helped the molecular sieving process. Reactive monoprotected tosylated PEG was added to the side arms along the polyether neckbone, confirmed by a real-time monitoring to ensure couplings proceed to completion. The protecting group employed was tetrahydropyran-1-yl (THP) acetal for temporary protection of the chain, being easily deprotected by mildly acidic conditions and simply removed by liquid–liquid extraction. The molecular sieving improved this process since the use of a membrane to separate the macromolecule from the building blocks promoted a maximized separation efficiency.^52^

**Scheme 7:**
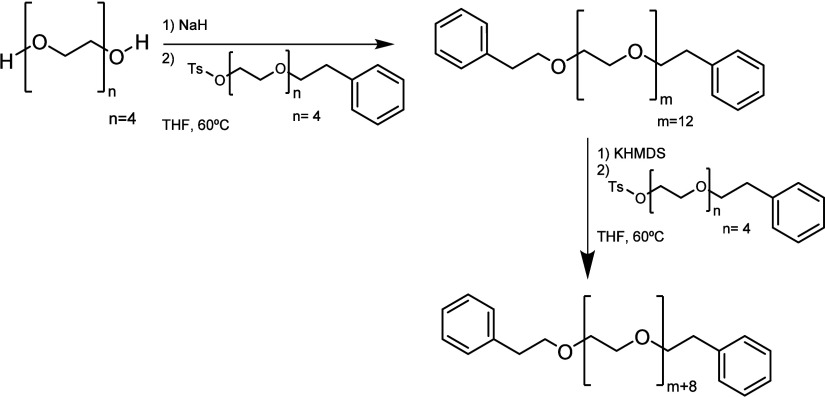
Synthetic Pathway Developed by Mikesell et al.

In 2021, a different methodology was elaborated to synthesize uniform PEGs with less steps; therefore, instead of doing the elongation of PEG in three steps, deprotection, deprotonation, and coupling, they achieved it in two steps (Scheme 7). To do so, a base-labile protecting group such as phenethyl was used. By employing this protecting group, the deprotonation step was not required.^53^ In 2022, the same base-labile approach was applied in solid-phase synthesis, having significant advantages over the acid-labile protecting group usually used (DMtr). This was due to the shortening of the synthesis cycles from three to two steps and higher efficiency for deprotection and coupling steps. The conversions were expected to be around 100% since the final product was uniform.^54^

Table 1 lists all of the synthetic pathways developed to obtain uniform PEGs. It is clear that the longer the PEG chain, the overall yield decreases significantly.^53^ The major cause of this phenomenon is that longer PEG oligomers are not easily available.^55^

**Table 1:** Uniform PEGs Synthetic Pathways, with a Focus on Overall Yields, Scalability, and Purity of the Polymers^a^

authors/reference	scale (g)	purity	PDI (polydispersity index)	PEG units	overall yield (%)
Fordyce et al.^36^	92	ND	ND	6	48
	230			8	50.3
Bömer et al.^37^	ND	ND	ND	9	81
Marshall et al.^38^	9.21	ND	ND	9	22.3
	1.55	ND	ND	12	4.6
Marshall et al.^39^	98.3	99.9%	ND	5, 10, 15, 20, 25	90–95
Nakatsuji et al.^40^	9.76	ND	ND	6	58
	2.1			8	55
Kinugasa et al.^41^	5.07	ND	ND	54	ND
Zada et al.^42^	83	ND	ND	8	84
	51.6			9	87
	30			11	60
Louseau et al.^43^	>10	ND	ND	6, 12, 15, 18, 24	>80
Burkett et al.^45^	ND	ND	ND	7–12	65–84
Ahmed and Tanaka^56^	1.6	insufficient	ND	7–44	>90
French et al.^35^	35–75	99.6–99.9%	1.00009	16	57
		98.9%	ND	32	ND
		98%	ND	48	ND
Gothard et al.^49^	1.7	ND	ND	10	70–73
				12	
Maranski et al.^57^	2–6	96.1–98.8%	ND	11–15, 19–22	<80
Zang et al.^17^	>100	assumed high	ND	8	76
				16	85
				24	81
				32	78
				40	73
				48	80
				56	81
				64	76
Li et al.^51^	14	assumed high	ND	7	87
	10			11	94
	10			15	93
	8			19	84
Xia et al.^58^	53	assumed high	ND	12	61
Wawro et al.^14^	ND	assumed high	ND	16	ND
Khanal and Fang^16^	0.023	ND	ND	8	82
	0.042			12	79
Dong et al.^52^	5.6	assumed high	ND	8	90
Mikesell et al.^53^	1.4	ND	ND	12	97
	1.765			20	86
	1.6			28	70
	0.436			36	25
	0.199			44	43
Eriyagama et al.^54^	ND	ND	ND	9	
				14	
				19	

a ND: not defined.

Even though the reported publications state that uniform PEG was synthesized, most lack PDI values to support this information and its confirmation is mainly done by mass spectrometry. The same happens with purity since the majority of the publications did not give the exact value. Moreover, the method developed by Eriyagama et al.^54^ clearly shows that the longer the PEG chain, the more difficult the reaction due to different PEG conformations leading to lower yields. However, several other reported studies were able to overcome this loss of yield, always ensuring high yields, being more prone to successful employment in industry.^17,39,43,52^

### 2.3 Monoprotection

**Scheme 8:**
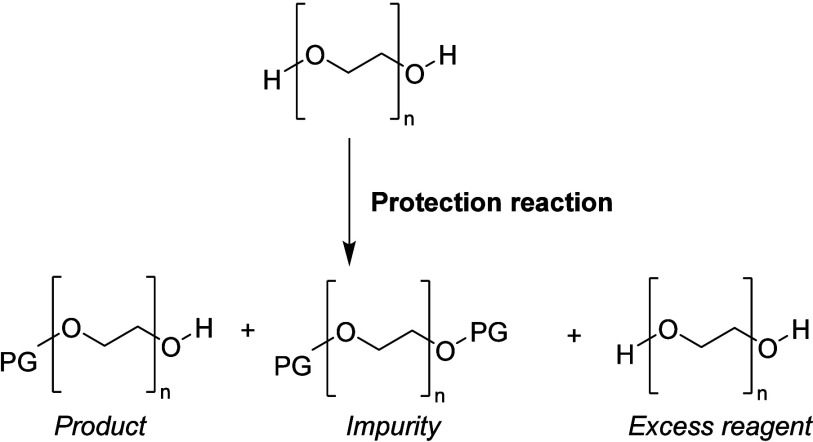
General Outcome of the Protection Reaction of PEG

Chemically modifying only one end of a symmetrical homobifunctional molecule like PEG is a challenging task. Moreover, preparing monoprotected derivatives of symmetrical bifunctional compounds using solution-based methods often results in mixtures of bisprotected, monoprotected, and unprotected products that are difficult to separate from one another (Scheme 8). As reported, conducting the monoprotection reaction of PEG usually promotes an isolated yield for monoprotected PEG between 66% and 80%.^46^

Several different methods are described in the literature for achieving this type of chemical modification. One of them is to add a large excess of a bifunctional molecule, but such conditions result in a mixture that consists of a large amount of unreacted starting materials, a small amount of bisprotected product, and only a minor amount of the desired product.^59^ Another approach is to use a large excess of the PEG to promote monoprotection; however, this also results in a mixture of unreacted starting material, bisprotected product, and the desired product, making it necessary to use other purification techniques to obtain the monoprotected product.^57^ Other approaches used to synthesize heterofunctional PEGs include ethylene oxide polymerization with an end-cap step using *p*-vinylbenzyl chloride and methacryloyl chloride. This approach starts with deprotonated 2-(*tert*-butyldimethylsiloxy)ethanol, which was prepared by reacting it with potassium naphthenylide. This alkoxide was then used to initiate polymerization with ethylene oxide. The resulting product was quenched with benzyl chloride, and the silyl group was subsequently removed to generate PEG monobenzyl ether. However, this process has several drawbacks since the starting raw material is not available and relies on a polymerization method that will produce disperse PEG.^60^

Later, another approach employed to overcome this challenge was made by Reel et al., where monobenzyl ether PEG was successfully synthesized using potassium hydride as base for the generation of the alkoxide initiator, which then reacts with ethylene oxide in THF to give high yields (95–99%) of pure PEG monobenzyl ethers. Afterward, the polymerization was quenched with Amberlyst IR-120(+) and filtered from the resin directly into cold diethyl ether to precipitate the product. However, this method presents certain limitations due to the utilization of ethylene oxide and high molecular weight PEGs, which enable easy precipitation, a phenomenon that is not observed with lower PEGs.^61^

Moreover, Ehteshami et al. developed another approach to synthesize monoprotected PEG through the use of batchwise ion-exchange chromatographic separation of charged and uncharged species. They started with NH_2_–PEG–NH_2_ and did the amine-based protection reaction. Afterward, to separate the monoprotected PEG from the bisprotected PEG, an ion adsorption column was used to retain monoprotected PEG and remove the bisprotected species.^6^

**Scheme 9:**
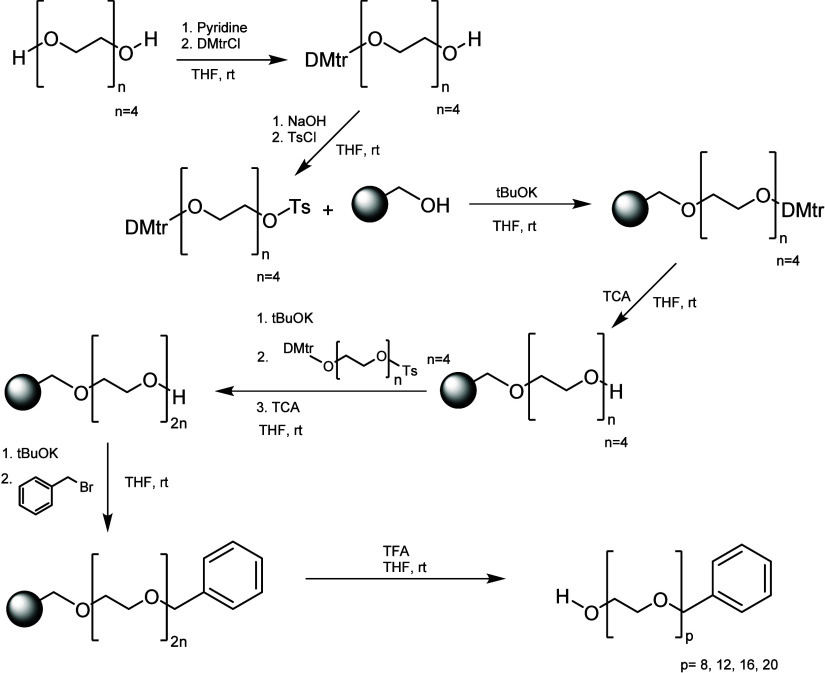
Solid-Phase Monoprotection of PEG

There have been attempts to address the challenge of isolating and purifying specific products. One alternative method involved creating solid-phase-based synthetic techniques that utilized temporary obstruction of functional groups in these compounds. In 2017, Khanal et al. proposed a solid-phase technology for PEG monoprotection with benzyl bromide utilizing Wang resin. This process involved purifying all intermediates through washing and yielded pure final products without the need for chromatography (Scheme 9).^19^

A different method was used to create a mixture of monoprotected PEG and bisprotected PEG by using the benzylate PEG chain. The mixture was then treated with inexpensive THP, which reacted only with monoprotected PEG. Complete removal of THP ethers was achieved through a transacetalization reaction between THP ethers and methanol or ethanol solvent, catalyzed with TsOH. After the deprotection step via acetal exchange, the THP ethers of methanol or ethanol were easily removed in vacuo due to their low boiling points. Finally, the hydrophilic PEG was removed by washing the solution with an aqueous NaCl solution to yield pure monoprotected product. The current process requires an additional step, which makes it less efficient.^44^

Despite several synthetic methodologies being applied to monoprotected PEG, none have been found to be completely optimal. Therefore, further research is necessary to identify the most efficient and effective method for monoprotected PEG synthesis.

## 3 Synthesis of PEG Derivatives

A substantial range of chemical modifications of PEG was tested, reported, and commercialized to provide conjugation of the polymer with small and macromolecular therapeutic agents, including drugs, oligo- and polypeptides, proteins (enzymes and antibodies), oligonucleotides, and biomaterials surfaces. Moreover, PEG has also been used as a cross-linker in cases in which bifunctional derivatives of PEG have been synthesized. Since PEG is bound to other molecules, it typically increases their solubility in aqueous media and yields improved circulation times in vivo; it is essential to develop PEG derivatives to allow possible connection between two defined length components.^25,62^

Usually, two different methods are employed for synthesizing PEG derivatives. The most used is ring-opening polymerization of EO from a heterobifunctional anionic initiator, and this is followed by termination with another functional moiety. However, as previously explained, these types of polymerizations have wide dispersity. The second method involves partial derivatization of PEO diols, followed by separation of the resultant statistical mixtures to isolate the targeted derivatives. It usually consists of the modification of the terminal hydroxyl groups of PEG through a series of reactions, followed by separation of the mono-, di-, and unsubstituted components, leading to several reaction steps with low yields and multiple isolations. Moreover, as the molecular weight of PEG increases, the chemical and physical differences among the mono-, di-, and unsubstituted products become even more reduced, resulting in more problems in isolation.^25^

### 3.1 First-Generation PEG Chemistry

The first and most straightforward strategy for the covalent attachment of PEG chains on proteins employs naturally occurring nucleophiles, amine or thiol groups, in the side chains of the amino acids lysine or cysteine. These were addressed using the electrophilic PEG derivatives described below, but because proteins contain multiple nucleophiles, it is frequently necessary to use too much PEGylation reagent to achieve reasonable conversions. Overall, the derivatives of the first-generation PEG chemistry end modify multiple residues in a protein, resulting in a heterogeneous mixture of protein–PEG conjugates.^63^

#### 3.1.1 Amino

Amino-terminated PEGs were the first target of PEGylation derivatives since they are the most represented groups in proteins, generally exposed to the solvent, and can be modified with a wide selection of chemical strategies. There are many approaches to add an amino end group to PEG: through alkylation, which maintains the positive charge of the starting amino group, or acylation, complemented by loss of charge.^64^ However, the cost of amino-terminated PEGs can be prohibitively expensive compared to their hydroxy-terminated counterparts, which may make them inaccessible for some laboratories, or limit large-scale synthesis and use.

**Scheme 10:**
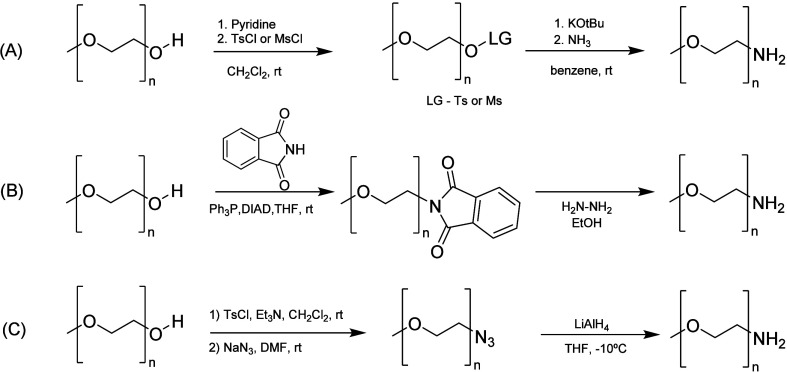
Procedures for the Preparation of PEG Derivatives with an Amino End Group (A) Leaving group approach; (B) Mitsunobu reaction; (C) sulfonate derivatives followed by reduction to amino end groups.

There are several published procedures for preparation of PEG derivatives with amino groups. The most common synthetic method involves the conversion of the hydroxyl group to a halide or sulfonyl ester (tosylate or mesylate) first, followed by reaction with an excess of ammonia (Scheme 10A). However, this synthetic pathway has as major drawback: the formation of secondary amine byproduct.^65^ Other approach is the conversion of PEG hydroxyl end groups to phthalimido end groups via the Mitsunobu reaction followed by the addition of hydrazine to afford amino-terminated PEGs (Scheme 10B).^66^ Moreover, a three-step strategy was developed involving conversion of the PEG hydroxyl end groups to azido end groups via the corresponding sulfonate or halide derivatives followed by reduction to amino end groups with either triphenyl phosphine (PPh_3_) or lithium aluminum hydride (LiAlH_4_) (Scheme 10C).^67^

However, the direct coupling of PEG–NH_2_ to activated protein was very challenging because these would react with the amines of the protein itself or another nearby protein molecule to yield intra- or intermolecular linkages. To overcome this issue, different derivatives were used for protein PEGylation of either the alpha or the epsilon amino groups. These chemistries generally contained PEG impurities, restriction to low molecular weights, unstable linkages, and lack of selectivity in modification. Examples of these first-generation PEG derivatives include PEG dichlorotriazine, PEG tresylate, PEG succinimidyl carbonate, PEG benzotriazole carbonate, PEG-nitrophenyl carbonate, PEG trichlorophenyl carbonate, PEG carbonyl imidazole, and PEG succinimidyl succinate.^8^

#### 3.1.2 Dichlorotriazine

**Scheme 11:**
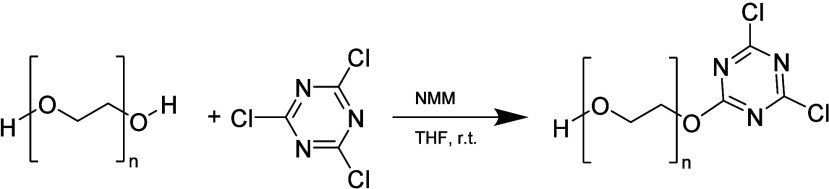
Synthesis of PEG Dichlorotriazine

The PEG dichlorotriazine derivative can react with numerous nucleophilic functional groups like lysine, serine, tyrosine, cysteine, and histidine. This reaction removes one of the chlorides and generates a conjugate, leaving a less reactive chloride that is not readily susceptible to further reactions with nucleophilic residues. However, this reactivity can lead to the undesired cross-linking of protein molecules that contain additional nucleophilic residues.^68^ The synthesis pathway described in the literature (Scheme 11) included the use of *N*-methylmorpholine (NMM) with cyanuric chloride (TCT) in anhydrous THF with PEG over the course of 3 h.^69^

#### 3.1.3 Tresylate

**Scheme 12:**
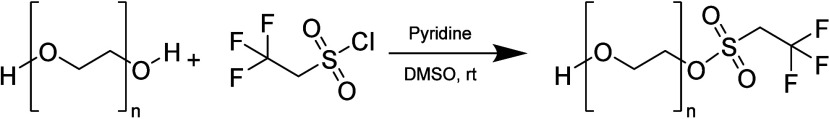
PEG Tresylate Synthesis

PEG tresylate (Scheme 12) was shown to be sufficiently reactive toward amino groups (more reactive than tosylates) and was considered useful as protein-modifying reagents.^68^ The synthetic pathway involves the reaction between tresyl chloride and PEG in DMSO with pyridine at room temperature.^70^

#### 3.1.4 Succinimidyl Carbonate

**Scheme 13:**
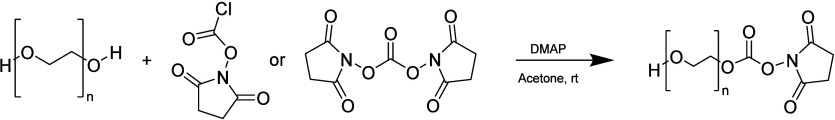
PEG Succinimidyl Carbonate Synthetic Pathway

Succinimidyl carbonate (Scheme 13) is one of the several functionalized PEGs with diverse molecular weights tested for PEGylation of proteins already employed in FDA-approved drugs such as Pegasys, PegIntron, and Asparlas. PegIntron is a covalent conjugate of interferon alfa-2b linked to a single unit of MW 12 000 PEG. Monomethoxy-PEGN-succinimidyl carbonate is subject to nucleophilic attack from several possible amino acid residues. It stands as the first-generation derivative for amine conjugation, and its synthesis involves the use of DMAP for hydroxyl end-group activation and *N*-succinimidyl chloroformate or *N*,*N*′-disuccinimidyl carbonate for reaction completion of the derivate.^71^

#### 3.1.5 Succinimidyl Succinate

**Scheme 14:**
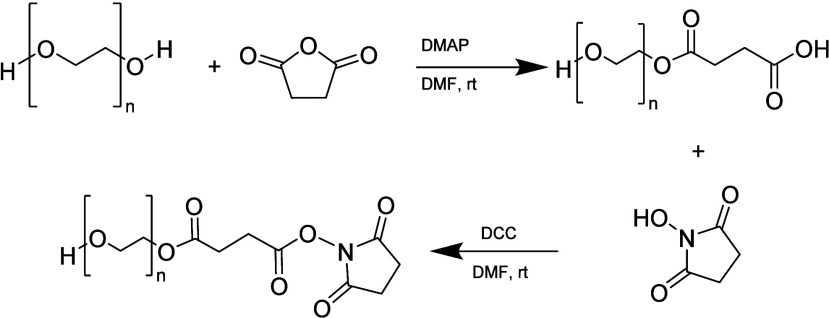
Synthetic Pathway of PEG Succinimidyl Succinate

PEG succinimidyl succinate (SS-PEG) is synthesized through the reaction of PEG with succinic anhydride, followed by activation of the carboxylic acid to the highly reactive succinimidyl ester (Scheme 14).

These derivatives are important since many biologically relevant ligands have been covalently attached to PEO through amide bonds via these succinimidyl ester intermediates. The first step comprises the synthesis of carboxylic PEG with the use of pyridinium dichromate (PDC) in DMF at room temperature with a yield of 58%^25^ or the use of DMAP as catalyst with succinic anhydride and TEA.^72^ Furthermore, carboxylic PEG is used as a precursor for the synthesis of succinimidyl PEG through the reaction of mPEG with succinic anhydride, followed by activation of the carboxylic with the combination of dicyclohexylcarbodiimide (DCC) and *N*-hydroxysuccinimide (NHS).^73^

Moreover, Oncaspar and Adagen, which are FDA-approved drugs, employ PEG succinate in polymer–protein conjugation. Adagen first started to use PEG dichlorotriazine; however, even though the remaining chlorine is not as electrophilic, it reacted to cause protein cross-linking. This is a nonspecific attachment process, so multiple units of PEG are attached to the protein; therefore, even though this PEG was utilized first by the company to study the drug, it is not what is used in the FDA-approved formulation. In the case of Oncaspar, the NHS group is displaced by nucleophilic amino acid units such as lysine, serine, cysteine, tyrosine, and histidine. The PEG ester and thioesters are highly susceptible to hydrolysis; thus, modification occurs primarily at the amines, forming amides.^74^

Furthermore, this derivate contains a second ester linkage after the conjugation reaction with a protein. This linkage is highly susceptible to hydrolysis after the polymer has been attached to the protein, possibly triggering a loss of benefits.^8^

The remaining PEG derivatives that produce urethane-linked proteins include *p*-nitrophenyl carbonate, trichlorophenyl carbonate, and carbonylimidazole, which are prepared by reacting chloroformates or carbonyl imidazole with the terminal hydroxyl group on PEG having much lower reactivity; therefore, it will not be further addressed.^8^

Although PEGylated protein drugs produced through the described methods are administered as a mixture of proteins with reduced biological activity, their increased half-life leads to a significant improvement in pharmacological potency compared to non-PEGylated drugs. Therefore, several PEGylated protein drugs have received FDA approval.^63^ However, first-generation processes have drawbacks, such as the presence of mixtures of isomers, diol contamination, unstable bonds, and changes in the bioactivity of some biomolecules.^75^

### 3.2 Second-Generation PEG Chemistry

The second-generation PEGylation chemistry has been designed to avoid the above-noted problems of diol contamination, restriction to low molecular weight mPEG, unstable linkages, side reactions, and lack of selectivity in substitution.

#### 3.2.1 Aldehyde

**Scheme 15:**
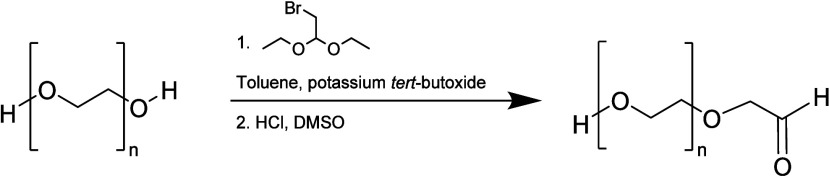
Synthetic Pathway of PEG Aldehyde

One of the first examples of second-generation chemistry is mPEG-propionaldehyde or PEG-aldehyde (Scheme 15), which are largely selective for the N-terminal α-amine because of the lower p*K*_a_ of the α-amine compared to other nucleophiles.^8^ PEG-aldehyde literature in synthesis reports that the first method exploited an oxidation reaction realized by heating PEG dissolved in a solution of acetic anhydride in DMSO. In the second route, bromo acetaldehyde diethyl acetal was added to a solution of PEG and potassium *tert*-butoxide in toluene in order to produce a PEG-acetal intermediate, which was treated with HCl to obtain the aldehyde derivative by acid hydrolysis.^76^

#### 3.2.2 Carboxylic Acids

For PEGylation of proteins, usually lysine, alpha, cysteine, histidine, arginine, aspartic acid, glutamic acid, serine, threonine, tyrosine and epsilon amino groups are most often conjugated with PEG. To conjugate with lysine residue, carboxylic acid-terminated PEG is most frequently used.^77^ Moreover, the carboxyl terminus of PEO can be activated by forming highly reactive succinimidyl esters, as previously mentioned, and the carboxyl end-group PEGs have applications in biomedical areas since they can be linked to substrates via ester bonds that can be hydrolyzed under certain conditions.^78^

**Scheme 16:**
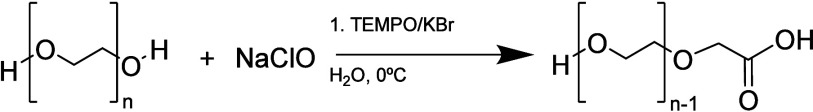
Synthesis of PEG-Carboxylic Acid

The synthesis includes the reaction of PEG with catalytic amounts of 2,2,6,6-tetramethyl-1-piperidineoxyl (TEMPO) and KBr as the regenerating oxidant in water (Scheme 16).^79^

#### 3.2.3 Vinyl Sulfone

**Scheme 17:**
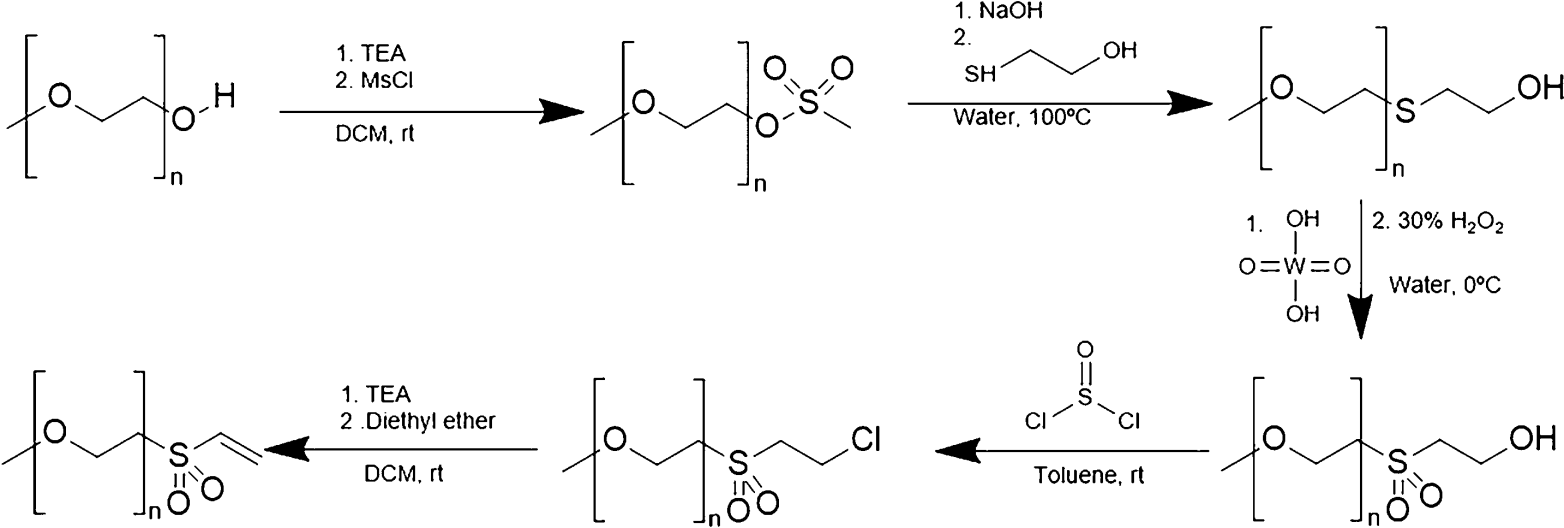
Synthesis of PEG-Vinyl Sulfone

PEG-vinyl sulfone (PEG-VS) reacts slowly with thiols to form a stable thioether linkage to the protein at slightly basic conditions (pH 7–8). However, the reaction proceeds faster if the pH increases. Although PEG-VS is stable in aqueous solutions, it may react with lysine residues at high pH levels. The synthesis of this compound involves multiple steps (Scheme 17). First, mPEG chloroethyl sulfone is synthesized by adding methane sulfonyl chloride (MsCl) and TEA to a solution of mPEG in dichloromethane for 20 h. In the second step, the mesylate reacts with β-mercaptoethanol in the presence of NaOH to form mPEG hydroxyethyl sulfide. In the third step, the sulfide is oxidized to produce mPEG hydroxyethyl sulfone with a tungstic acid solution and H_2_O_2_, which is later converted to chloride by reacting with thionyl chloride. The chloroethyl sulfone (CES-PEG) is easily converted to vinyl sulfone (VS-PEG) by reacting with various bases (e.g., TEA).^80^

PEG-VS is stable in water and reacts slower with thiols to form a thioether linkage to protein. The reaction rate can be increased by increasing the pH level to slightly primary conditions (pH 7–8).^8^

#### 3.2.4 Maleimide

**Scheme 18:**
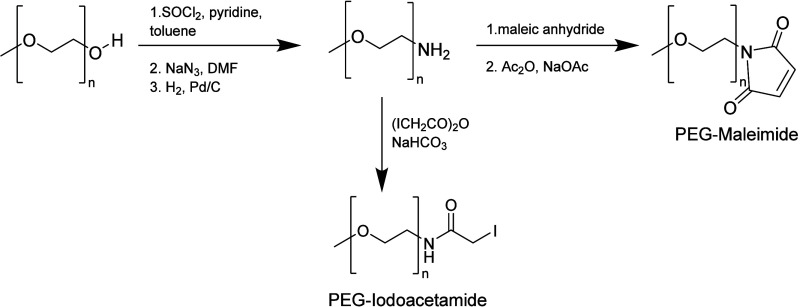
Synthesis of PEG-Maleimide and PEG-Iodoacetamide

PEG-maleimide (PEG-Mal) is synthesized by first treating amine and then reacting the product with maleic anhydride to generate the intermediate maleamic acid. This is dehydrated with acetic anhydride and sodium acetate to produce the maleimide (Scheme 18).^81^

The reaction of PEG-maleimides with thiols is one of the best known site-specific, simple, and quantitative protein chemical modification reactions. However, its synthesis uses multiple steps with a lower level of functionalization.^82^ In addition, PEG-Mal is more responsive to thiols even under acidic conditions (pH 6–7); however, it is not stable in water and can undergo ring opening or the addition of water across the double bond. While the thioether linkage between PEG-Mal and protein is stable, one of the amide linkages can be slowly cleaved by hydrolysis.^8,68^

#### 3.2.5 Iodoacetamide

PEG-iodoacetamide (PEG-IA) is synthesized by treating the amine with iodoacetic anhydride in dioxane containing sodium bicarbonate. This results in the formation of PEG-iodoacetamide (Scheme 18).^81^ It reacts slowly with free thiols by nucleophilic substitution, creating a stable thioether linkage. However, the reaction should be performed in a slight molar excess of PEG-IA in a dark container to limit the generation of free iodine that may react with other amino acids.^8^

#### 3.2.6 Orthopyridyl Disulfide

**Scheme 19:**
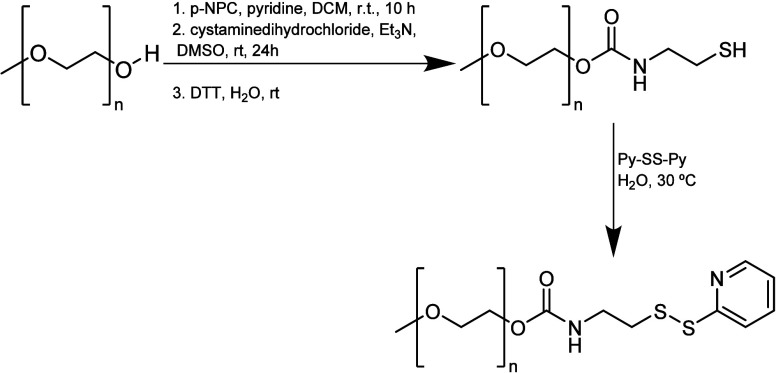
Synthetic Pathway To Obtain PEG-Orthopyridyl Disulfide

PEG-orthopyridyl disulfide (mPEG-SS-Py) was synthesized according to the following procedure (Scheme 19): (1) activating PEG with *p*-NPC (C_7_H_4_ClNO_4_), (2) reacting with cystamine dihydrochloride in the presence of Et_3_N, (3) reducing with DTT (C_4_H_10_O_2_S_2_) to yield PEG-SH, and (4) exchanging with 2,2′-dipyridyldisulfide (Py-SS-Py). All of the steps had yields above 80%.^83^

Furthermore, mPEG-SS-Py reacts specifically with sulfhydryl groups under both acidic and basic conditions (pH 3–10) to form a disulfide bond with the protein. Disulfide linkages are also stable, except in a reducing environment when the linkage is converted to thiols.^8,68^

#### 3.2.7 Hydrazide

**Scheme 20:**
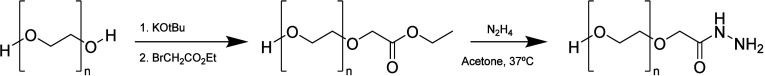
Synthesis of PEG-Hydrazide

Direct coupling of PEG–NH_2_ to activated protein carboxylic groups is not possible because these would react with the amines of the protein itself or of another nearby protein molecule to yield intra- or intermolecular linkages. To overcome this issue, PEG hydrazide is used, which is reactive at low pH as an amino donor.^84^ The hydrazone linkage can be converted into a stable alkyl hydrazide and Schiff’s base to a secondary amine by reducing with sodium cyanoborohydride. By adjusting the pH to around 5, it is possible to selectively modify the carbohydrate sites of the protein using PEG-hydrazides. This results in multiple attachment sites, but the modification is specific to the carbohydrate sites of the protein. The synthesis of this derivate is described in Scheme 20. The first step comprises carboxymethylation of PEG with a α-haloacetic acid. This is best accomplished by displacement of the bromide in ethyl bromoacetate with PEG-alkoxide, followed by reaction of the ester with hydrazine.^65^

#### 3.2.8 Thiol

**Scheme 21:**
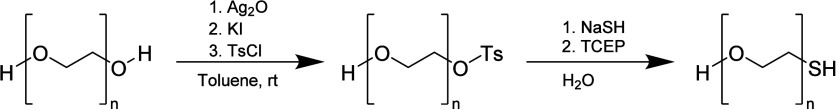
Synthetic Approach for the Synthesis of Thiol-Terminated PEG

Thiol-terminated PEG is widely used in the functionalization of nanoparticles by increasing their stability and their hydrophilicity and thus reduces their toxicity in biological systems.^85,86^ Furthermore, this derivative has been widely used in gold nanoparticles since the surface gold atoms bind to the thiol group of the PEG, being an efficient PEGylation agent of these types of drug delivery.^87,88^ The described synthesis (Scheme 21) consists of the preparation of tosyl-PEG. To achieve a selective monotosylation, an excess of symmetric diols was employed using a stoichiometric amount of tosyl chloride in the presence of Ag_2_O and a catalytic amount of KI.^89^ Afterward, the tosylated PEG is reacted with an excess of sodium hydrosulfide hydrate in water, providing the product PEG-SH.^86^

#### 3.2.9 Methoxy

Methoxy-PEG (mPEG) is one of the most commonly used PEG derivatives in various biomedical and pharmaceutical applications due to its biocompatibility, hydrophilicity, and low immunogenicity. This derivative has several advantages over other PEG derivatives, including lower immunogenicity and improved stability. However, its synthesis is far from straightforward. Regular PEGs are very complex mixtures of homologues as a result of polymerization. Although many synthetic strategies have been developed for mPEGs since 1939, it is of great importance to develop efficient and scalable processes for mPEGs, especially fully functionalized mPEGs which can be directly used in biomedical research and development.^58^ The most common approach to synthesize this derivate involves the use of sodium methoxide (NaOMe) in methanol followed by sulfuric acid (H_2_SO_4_) in water.^33,58^ However, to ensure that only one extremity of PEG reacts, initial protection, deprotection, and activation of the hydroxyl group is necessary. Other approaches such as solid-phase synthesis^16,47^ and macrocyclization^17,33^ have been applied to effectively synthesize mPEGs selectively.

#### 3.2.10 Azido

**Scheme 22:**
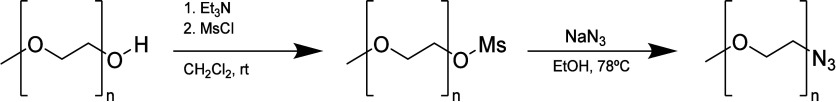
Synthetic Pathway of Azido-Terminated PEG

Lastly, azido-terminated PEGs are also employed in the PEGylation of bioconjugates. Its synthesis involves two overall steps: Mesylation of mPEG with mesyl chloride and TEA, obtaining the product mPEG-Ms that is then reacted with sodium azide in excess, providing the azido-terminated PEG (Scheme 22).^90^

In general, end-group functionalization of PEG has been conventionally conducted through esterification or etherification of the hydroxyl moiety. This has several disadvantages as the esterification reaction is a reversible process and complete conversion can be obtained only under extreme conditions (anhydrous, excess reactant, elevated temperature), and that ester linkage is incompatible with primary amine moieties due to possible amidation reactions. On the other hand, the etherification reaction of PEG involves the use of strong bases (e.g., NaH) and might lead to PEG chain scission. For this reason, Shi et al. carried out studies that used carbamate linkages for the synthesis of PEG–NH_2_ and further generalized them for various PEG derivatives.^91^

#### 3.2.11 Branched Structures

**Figure 3:**
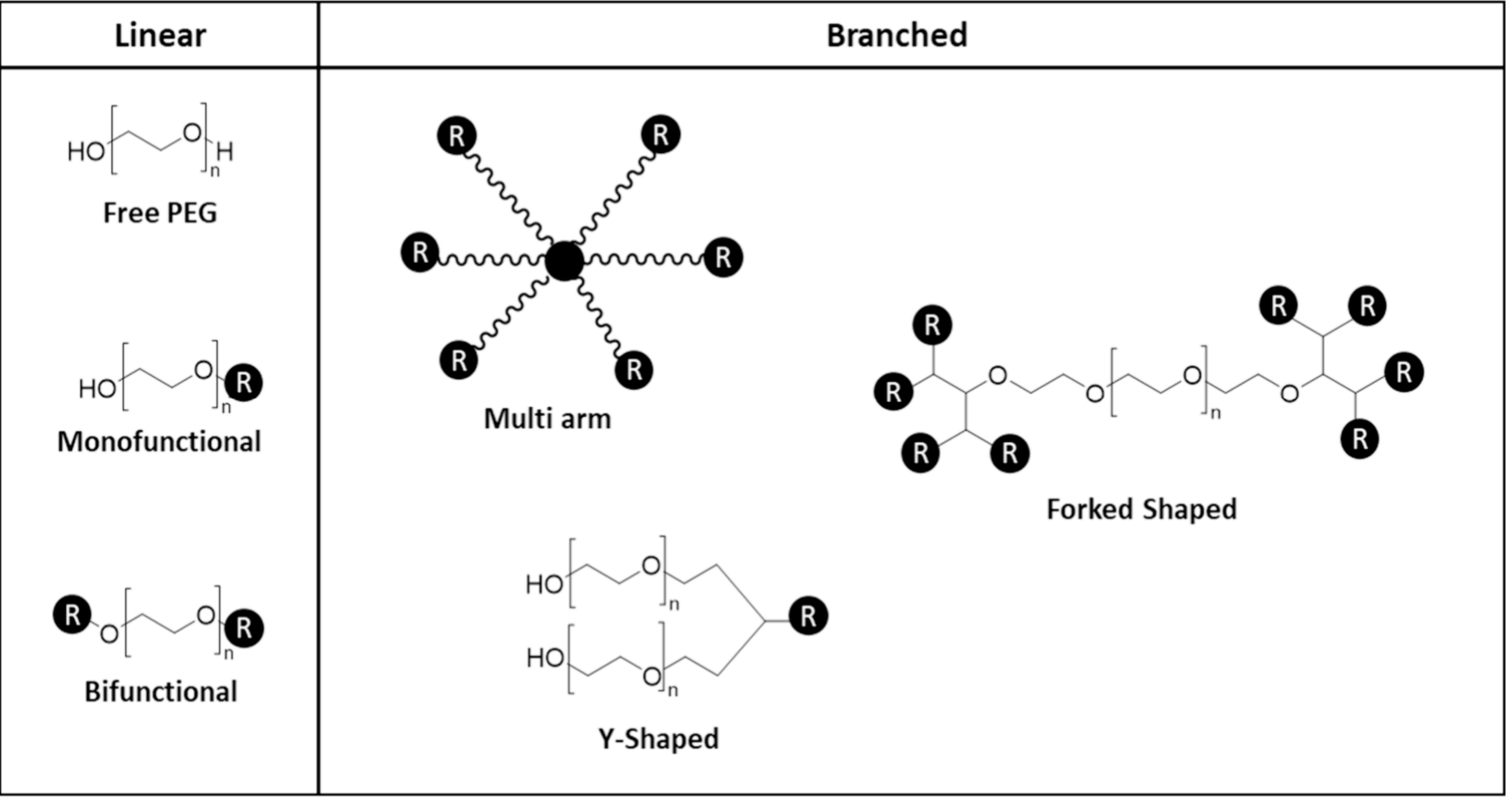
Possible branched PEG structures.

Furthermore, this generation also led to the preparation of branched PEGs of increased molecular masses (>40 kDa), which shields the protein surface better than a linear PEG of the same size as it is more effective in protecting the conjugated protein from proteolytic enzymes and antibodies.^75^ The branched structures can include multiple arrangements as seen in Figure 3.

These branched structures are quite appealing from an industrial standpoint. Recent studies have showcased the versatility of branched PEGs and their suitability for polymer therapeutics. Furthermore, the overall structure of branched PEGs suggests biocompatibility for several functional groups, with each group being contingent on the respective linker.^92^ For example, Liu et al. modified DSPE nanocarriers with mPEG using linear and branched structures and concluded that the branched modified nanocarriers had a good circulation time with great potential in avoiding the enhanced blood clearance phenomenon, improving the antitumor effect.^93^ Even though these branched structures are often associated with lower viscosities, Fee et al. analyzed the molecular sizes of linear and branched PEGylated proteins and concluded that there is no difference in the hydrodynamic volumes or viscosity of proteins PEGylated with the linear and branched forms of PEG regardless of the PEGylation extent. Nonetheless, a longer in vivo circulation half-life was observed for branched-PEG proteins, which were more effective at masking the protein surface when compared with linear PEG proteins.^94^ Frey et al. studied the synthesis of well-defined random copolymers, specifically P(EO-*co*-AGE)s, created through anionic ring-opening polymerization of ethylene oxide and allyl glycidyl ether (AGE). To achieve a random distribution of the comonomers, the reaction conditions were optimized to minimize allyl isomerization while maintaining a reasonable polymerization rate. This random copolymer structure is highlighted as being crucial for producing multifunctional PEG derivatives systematically without structural inhomogeneity. The modular synthetic approach facilitated the production of multifunctional PEG derivatives, including hydroxy, carboxy, and amino derivatives, while maintaining narrow molecular weight distributions with PDI values between 1.04 and 1.19. These derivatives were used for various conjugation purposes, demonstrating the versatility and efficiency of the synthetic platform.^95^

The synthetic pathway of these branched structures is very similar to dendrimer creation. The development of new branched, high molecular weight multimeric PEG-based systems (MultiPEGs) starting with inexpensive commercial PEG moieties assembled in a divergent dendrimeric way has been proposed as a synthetic approach. These novel compounds are created by selectively and briefly protecting only one of the two initial reactive ends of smaller commercial bifunctional PEGs. The assembly of PEG units with the necessary linkers by subsequent condensation processes using various synthetic methods is prevented by activation of the leftover hydroxylic groups.^96^

### 3.3 Challenges and Future Remarks

The molecular weight of PEG for therapeutic use is usually limited to no more than 10 kDa to ensure complete clearance since above this size the molecules cannot be effectively degraded by the liver, causing potential PEG accumulation in these organs and increasing the risk of toxicity. Hence, the solution to overcome this drawback is to synthesize biodegradable PEGs by introducing biologically degradable functional groups into PEG structures as ester bonds, amide bonds, disulfide bonds, carbonates,^97,98^ and vinyl ethers, which have been incorporated into PEG backbones.^99^

As a result of the many PEGylated drug’s improved pharmacokinetics and pharmacodynamics, PEG derivatives have revolutionized the pharmaceutical industry. In order to improve the therapeutic solubility and lessen the immunogenicity, the first-generation PEGylated drugs, created in the 1990s, employed linear polyethylene glycol molecules that were effective at increasing the medication efficacy and lowering the adverse effects, but their short half-lives made them ineffective.^100^

The second-generation PEGylated derivatives were created to address some of the drawbacks of the first-generation compounds. These are perfect for sustained-release formulations because they have enhanced drug-release characteristics and longer half-lives. Additionally, it has been demonstrated that second-generation PEGylated compounds enhance the therapeutic index of several medications, enabling the administration of greater doses. Currently, attempts to increase the efficacy of drugs are still under development. These intend to tackle critical problems with PEGylation, such as the size and position of PEG molecules on the conjugates that can affect their properties, PEG dispersity index, degree of PEGylation, and PEGylation site specificity.^77^

Overall, PEGylation has emerged as a crucial strategy in creating innovative drug formulations and has successfully helped numerous PEGylated medications reach the market. Future advancements in PEGylated technology and the creation of even more potent PEGylated substances are anticipated due to continuous research and development.

## 4 Characterization

PEG characterization poses several challenges due to the unique properties of this polymer as the lack of chromophores makes common direct detection challenging in techniques like UV–vis spectrometry, which relies on the presence of chromophores for detection. Moreover, since PEG often exists as a mixture of oligomers with different molecular weights and chain lengths, analysis is more challenging and necessitates the development of techniques that can resolve and quantify individual components within the mixture. This dispersity can complicate the analysis, particularly when trying to achieve accurate and reproducible measurements.^101,102^

### 4.1 High-Performance Liquid Chromatography (HPLC)

Accurate analysis and determination of the molecular weight (MW) of polyethylene glycols (PEGs) is extremely important, particularly for medium and large MWs. For this reason, researchers have explored the use of HPLC techniques, namely, reversed-phase HPLC (RP-HPLC) and normal-phase HPLC (NP-HPLC), for PEG analysis. Sun et al. examined both HPLC phases using derivatized PEGs with dinitrobenzoyl chloride. They discovered that the hydroxyl group of solvents in normal-phase HPLC played a crucial role in resolving and retaining PEG oligomers. Furthermore, normal-phase HPLC showed better resolution for high molecular weight PEG oligomers than RP-HPLC.^103,104^ Cho et al. confirmed this finding, stating that NP-HPLC with an amino-bonded silica stationary phase provided ease in analyzing high molecular mass PEG samples.^105^

Moreover, the temperature effect on the retention time was studied, and it showed that the separation of PEG polymers can be realized by using thermal gradients using an isocratic gradient; however, this was only verified for PEGs with MW < 2000 Da.^106^

### 4.2 Detectors

**Figure 4:**
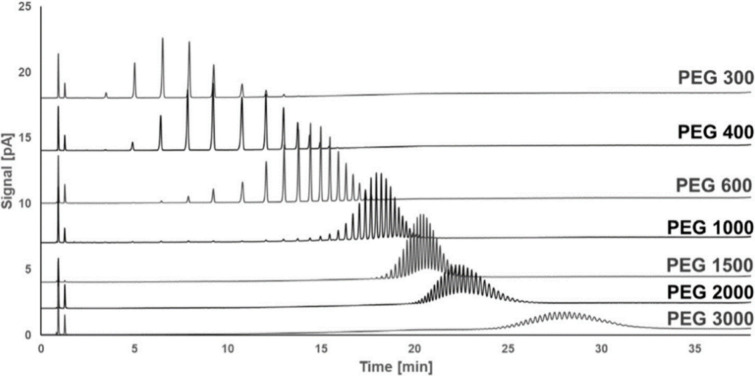
Separation of different PEGs using HPLC-CAD. Adapted with permission from ref (109). Copyright 2018 Elsevier.

The detection of native PEGs is difficult due to the lack of chromophores, which makes tedious and time-consuming derivatization procedures necessary. To overcome this challenge, other techniques such as ELSD (evaporative light scattering detection) can be used. Brinz and Holzgrabe used a simple linear water/methanol gradient and a Waters XTerraRP-18 (250 × 4.6 mm, 5 m particle size; Milford, MA, USA) analytical column. This method was able to separate PEGs up to an average molar mass of 1500 with acceptable resolution values. However, it failed to be applied to PEGs with higher chain lengths.^107^ Later, an efficient technique was developed using a RP-HPLC gradient performed on a C18 column with a binary gradient of acetonitrile and water and a nebulization chamber at 30 °C. This method allowed for the separation of PEGs in the molecular weight range between 400 and 4000 according to the number of ethoxylate units.^108^ Holzgrabe also developed an analytical reversed-phase HPLC method coupled to a charged aerosol detector (CAD) that could characterize disperse PEG. In this case, the mobile phases were water with 0.1% (v/v) formic acid and acetonitrile in a YMC-Pack Pro, a RP-18 column. Up until PEG1500 it was possible to completely separate into their respective oligomers, but for higher molecular weight polymers (PEG2000 and PEG3000) with higher dispersity, a decrease in resolution was encountered (Figure 4).^109^

Furthermore, a low amount of PEG impurity is highly desired to obtain uniform and high-quality monofunctional PEG products, which can be challenging to characterize. To this end, Barman et al. developed a reversed-phase liquid chromatographic method with ELSD to determine polyethylene glycol impurities in monofunctional PEG. It included an Altima C-18 column from Alltech that effectively separated PEG from M-PEG. Temperature is a significant factor in separating PEG from M-PEG at an optimum value of 55 °C. The isocratic composition of the binary mixtures is 50:50 water–acetonitrile for M-PEG 200 to M-PEG 1000 samples and 55:45 water–acetonitrile for M-PEG 2000 and M-PEG 3000 samples at a flow rate of 1 mL/min.^110^

LC-MS/MS is used to characterize PEG and PEGylated proteins. However, due to the heterogeneous nature of this polymer, the direct measurement of PEG or PEGylated proteins with LC-MS/MS has proven challenging. Recently, it was demonstrated that if PEG is induced to dissociate in the ionization source (in-source CID) of the mass spectrometer some PEG specific “daughter” ions can be generated and used to identify and quantify this polymer.^111^

PEG can also be measured with HPLC coupled with a refractive index (RI) detector. Although it has been reported as a method with low sensitivity, it has been successfully employed to separate PEG from the PEGylated protein.^112,113^

### 4.3 Mass Spectrometry

Since PEGylation converts a uniform protein into a dispersed macromolecule, the analysis of such samples by electrospray ionization MS (ESI-MS) becomes more challenging due to the formation of multiple charge states with different types of charges (protons, metal ions, ammonium ions) and the presence of unreacted PEG and PEG chains from degraded conjugate, which results in spectra containing several partially overlapping oligomeric distributions. To overcome this problem, Wesdemiotis et al. developed a complex analytical approach that can help provide a more comprehensive characterization of PEGylation products (UPLC-ISD-IM-MS approach). The UPLC dimension allowed separation based on polarity combined with shape/charge ion mobility (IM) dispersion with ion fragmentation (ISD) and mass analysis (MS).^114^

LC-MS/MS is used to characterize PEG and PEGylated proteins. However, due to the heterogeneous nature of this polymer, the direct measurement of PEG or PEGylated proteins with LC-MS/MS has proven challenging. Recently, it was demonstrated that if PEG induced dissociation in the mass spectrometer’s ionization source (in-source CID), some PEG-specific “daughter” ions can be generated and used to identify and quantify this polymer.^111^

**Figure 5:**
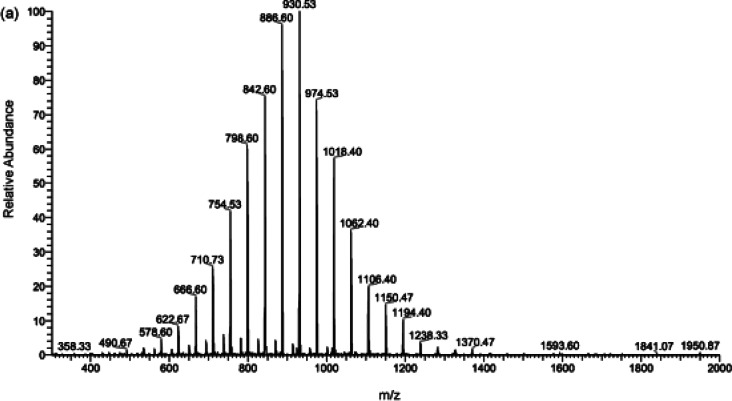
Mass spectra from a M-PEG750 sample obtained by LC-MS. Adapted with permission from ref (110). Copyright 2009 Elsevier.

While uniform PEG typically displays only one single molecular mass, the mass spectrum standards for disperse PEG can be found in Figure 5, thereby highlighting the characterization challenges.

In addition, matrix-assisted laser desorption ionization time-of-flight mass spectrometry (MALDI-TOF/MS) can also be used to characterize the monodispersity characteristic of PEG since it has an optimal degree of oligomer resolution. For this method it is important to first select the matrix, cationization agent, and solvent to optimize the effect on the ionization efficiency of the polymer. Wang et al. concluded that PEG-6000 has better compatibility with tetrahydrofuran and presented higher intensity signals using a DCTB matrix with a YAG laser at 335 nm and a pulse frequency of 40 Hz with an acceleration voltage of 20 kV in the positive ion mode.^108,115^ Many published works on the synthesis of uniform PEG rely on the use of this method to confirm the polymer dispersity.^14−17,35,41,42,45,51−53,57,58,116^ Moreover, comparing with other techniques, this is the one that can provide more useful information about polymer end-group analysis. PEG can be easily ionized via adduct formation with alkali metal ions due to the oxygen-rich nature of this polymer.^117^

### 4.4 Supercritical Fluid Chromatography (SFC)

Another approach being currently pursued to determine the accurate molecular weight of PEG is SFC, where it is possible to separate polymers molecularly. SFC is a powerful technique; however, the detection process in SFC is complex compared with that in normal liquid chromatography because for SFC the detector must be capable of operating at high pressures. Moreover, ultraviolet (UV) detectors are often used for SFC; however, PEGs do not have UV absorbance. Therefore, an ELSD detector needs to be used for the detection of PEGs, but its ability in the case of quantitative analysis of molecular weight has not yet been established.^118^

### 4.5 Size Exclusion Chromatography (SEC)

SEC is the most popular technique for size separation of polymers, and it is widely spread in polymer research laboratories. The PDI value is given by this analysis; however, the literature lacks SEC analysis to characterize uniform PEGs. From the previous literature describing uniform PEG synthesis, only two studies used SEC to characterize their polymer.^37,52^ Therefore, Bohn et al. analyzed several reported published procedures for the preparation of uniform PEG and analyzed them with this method, thus allowing an unambiguous comparison of the different procedures. It was proven that SEC is a powerful analytical tool that can monitor the reaction process, validate final purity, and provide PDI values.^34^ Despite all of the advantages, when analyzing ultrahigh-MW polymers, possible shear degradation and/or late elution of high-MW components can affect efficient size separation. In these cases, additional requirements are needed as the choice of a column with large particles and pore sizes, low flow rate, and the concentration of the sample should be below c* (c* = 1/[η]), where [η] is the intrinsic viscosity calculated by Mark–Houwink constants for a given MW.^119^

### 4.6 Nuclear Magnetic Response (NMR)

Lastly, NMR is widely used for polymer characterization, providing elucidation of the polymer structure and not dispersity.^14−17,35,41,42,45,51−54,56,58,116^ Besides the structure, NMR is used to calculate the purity and can be used to follow the synthetic pathways.

Overall, Table 2 presents a summary of PEG characterization methods described in the literature. For PDI values, MALDI-TOF, SFC, and SEC can be used, while for purity assay, the methods used include HPLC, GC, NMR, LC/MS, MALDI-TOF, and SEC.

**Table 2:** Characterization Methods of PEG

purpose	characterization methods
PDI	MALDI-TOF, SEC, SFC
purity assay	HPLC, GC, NMR, LC-MS, MALDI-TOF, SEC

## 5 Applications

### 5.1 Drug Delivery

#### 5.1.1 PEGylation

The most employed pharmaceutical application is in PEGylation, which arose in late 1970s as a popular technique used to enhance the therapeutic efficacy of drugs.^120^ PEG was stated as the perfect candidate for this purpose since it can be modified chemically by introducing different functional groups,^121^ easing the covalent or noncovalent attachment of PEG chains to therapeutic agents like small molecule drugs, peptides, and nucleic acids, thereby optimizing the pharmacokinetics and pharmacodynamics of drugs by improving the stability, reducing nonspecific protein absorption and macrophage uptake, and prolonging the circulation time through its “stealth effect”. Therefore, this technique has been extensively used in the pharmaceutical industry to enhance the therapeutic efficacy of drugs, making it a valuable tool for drug delivery.^9^

Furthermore, the use of PEGylation to achieve long-term drug action is because the increased molecular weight and hydrodynamic size of PEGylated drugs reduces renal filtration, leading to a longer circulation time and a decrease in dosing frequency, which makes this approach valuable in robustness and efficacy. However, PEGylation also presents some disadvantages. The polydispersity of conventional PEG has drawbacks in the synthesis and purification of PEGylated drugs and can result in heterogeneity that may compromise their reproducibility.^9^ Additionally, the nondegradable nature of PEG may elicit an immunogenic and antigenic response that can significantly impact the therapeutic efficacy and lead to unwanted side effects.^122^ However, this immunogenicity reaction can be decreased by using uniform PEGs instead of disperse PEGs.^123^ This was studied by Wang et al., who compared PEGylated proteins with uniform and disperse PEG and concluded that the monodispersity and biodegradability of the uniform PEGylation agents were successfully passed on to the peptides and proteins with enhanced hydrophilicity and reduced immunogenicity.^124^ However, even though uniform PEGs are expected to have lower immunogenicity, the FDA-approved pharmaceuticals mainly include disperse PEG (Table 3).

**Table 3:** FDA-Approved PEGylated Pharmaceuticals

tradename	chemical structure	clinical use	year of FDA/EMA approval	PEG length	type	PEG type
Adagen	adenosine deaminase	severe combined immunodeficiency disease (SCID)	1990	5 kDa	protein	monomethoxypolyethylene glycol
Oncaspar (pegasparaginase)	PEGylated l-asparaginase	treatment of leukemia	1994	5 kDa	protein	monomethoxypolyethylene glycol
Doxil (doxorubicin)	liposomal	ovarian cancer, multiple myeloma	1995	2 kDa	liposomal	monomethoxypolyethylene glycol
Pegintron (peginterferon alfa-2b)	PEGylated interferon alpha-2b	treatment of chronic hepatitis C, melanoma	2000	12 kDa	protein	monomethoxypolyethylene glycol
Neulasta (pegfilgrastim)	PEGylated recombinant methionyl human granulocyte	treatment of severe neutropenia induced by cancer	2002	20 kDa	protein	polyethylene glycol
Somavert (pegvisomant)	PEGylated human growth hormone receptor antagonist	treatment of acromegaly	2002	4 kDa	macromolecular	polyethylene glycol
Pegasys (peginterferon alfa-2a)	PEGylated interferon alpha-2a	treatment of chronic hepatitis C and hepatitis B	2002	20 kDa	protein	bismethoxypolyethylene glycol
Macugen (pegaptanib)	PEGylated angiogenic agent	treatment of neo-vascular age-related macular	2004	20 kDa	macromolecular	monomethoxypolyethylene glycol
Mircera (methoxy polyethylene)	PEGylated erythropoietin’s form	treatment of anemia associated with chronic kidney disease	2007	30 kDa	protein	monomethoxypolyethylene glycol
Cimzia (certolizumab pegol)	PEGylated monoclonal antibody specific to tumor necrosis factor alpha	treatment of severe rheumatoid arthritis and Crohn’s disease	2008	40 kDa	antibody	polyethylene glycol
Krystexxa (pegloticase)	PEGylated uricase	treatment of severe gout	2010	10 kDa	protein	monomethoxypolyethylene glycol
Asclera (polidocanol)	dodecyl alcohol	varicose veins	2010	400 Da	small molecular drug	polyethylene glycol
Sylatron	interferon α-2b	melanoma	2011	12 kDa	protein	monomethoxypolyethylene glycol
Omontys (peginesatide)	PEGylated erythropoietic agent	treatment of anemia associated with chronic kidney disease in adult patients on dialysis	2012	20 kDa	protein	monomethoxypolyethylene glycol
Plegridy (peginterferon beta-1a)	PEGylated interferon beta-1a	treatment of patients with relapsing forms of multiple sclerosis	2014	20 kDa	protein	monomethoxypolyethylene glycol
Movantik (naloxegol)	PEGylated naloxol	treatment of opioid-induced constipation	2014	339 kDa	small drug	monomethoxypolyethylene glycol
Adynovate	PEGylated antihemophilic factor	treatment of hemophilia A	2015	20 kDa	protein	polyethylene glycol
Onivyde (irinotecan liposomal)	liposomal	pancreatic cancer	2015	2 kDa	liposomal	monomethoxypolyethylene glycol
Jivi (damoctocog alfa pegol)	recombinant antihemophilic factor	hemophilia A	2017	30 kDa	protein	monomethoxypolyethylene glycol
Rebinyn	recombinant coagulation factor lX	hemophilia B	2017	40 kDa	protein	monomethoxypolyethylene glycol
Palynziq (pegvaliase-pqpz)	PEGylated recombinant phenylalanine ammonia-lyase	treatment of phenylketonuria	2018	20 kDa	protein	mono-*N*-hydroxylsuccinimide (NHS) polyethylene glycol
Udenyca (pegfilgrastim-cbqv injection)	G-CSF	infection during chemotherapy	2018	20 kDa	protein	monomethoxypolyethylene glycol
Revcovi (elapegademase-lvlr)	recombinant adenosine deaminase	ADA-SCID	2018	20 kDa	protein	monomethoxypolyethylene glycol
Fulphila (pegfilgrastim-jmdb)	G-CSF	infection during chemotherapy	2018	20 kDa	protein	monomethoxypolyethylene glycol
Asparlas (calaspargase pegol)	l-asparaginase	leukemia	2018	5 kDa	protein	monomethoxypolyethylene glycol
Esperoct (turoctocog alfa pegol)	recombinant antihemophilic factor	hemophilia A	2019	40 kDa	protein	polyethylene glycol
Ziextenzo (pegfilgrastim-bmez)	G-CSF	neutropenia	2019	20 kDa	protein	polyethylene glycol
Nyvepria (pegfilgrastim-apgf)	G-CSF	neutropenia	2020	20 kDa	protein	monomethoxypolyethylene glycol
Besremi (ropeginterferon alfa-2b)	interferon	polycythemia vera	2021	40 kDa	protein	monomethoxypolyethylene glycol
Skytrofa (lonapegsomatropin)	human growth hormone	growth hormone deficiency	2021	10 kDa	protein	monomethoxypolyethylene glycol
Empaveli (pegcetacoplan)	pentadecapeptide	paroxysmal nocturnal hemoglobinuria (PNH)	2021	40 kDa	protein	polyethylene glycol
Rolvedon (eflapegrastim-xnst)	Spectrum Pharmaceuticals	neutropenia	2022	3.4 kDa	protein	polyethylene glycol
Stimufend (pegfilgrastim-fpgk)	G-CSF	neutropenia	2022	20 kDa	protein	monomethoxypolyethylene glycol
Fylnetra (pegfilgrastim-pbbk)	G-CSF	neutropenia	2022	20 kDa	protein	monomethoxypolyethylene glycol

Even though it is challenging to synthesize a uniform PEG with a molecular weight above 3000 Da, there are approved drugs with lower molecular weight PEGs. An example is Asclera, where a monododecylated disperse PEG mixture was used with a PEG moiety average MW of 400 Da. Recent studies have shown that the drug involves disperse PEG being a very complex mixture, which complicates the production, quality control, clinical application, therapeutic efficacy, and safety because each component in polidocanol would have quite different physicochemical properties and biological effects. In fact, adverse effects such as pain, inflammation, and skin pigmentation were found in polidocanol-treated patients, which may be related to the unwanted components in polidocanol. A comparative study between uniform polidocanols and disperse polidocanol showed several advantages to the use of uniform PEG, such as improved cytotoxicity toward the targeted human umbilical vein cells, dramatically improved drug quality, efficacy, and safety, and convenient ways to fine tune the physicochemical and biological properties.^125^ Later, in 2017, Yu et al. confirmed the previous data through the development of a macrocyclic sulfate-based synthesis strategy of uniform polidocanols, their sulfates, and methylated derivatives for a comparative study of uniform and disperse polidocanols. Through this study, it was found that polydispersity in PEGs can downgrade the purity, bioactivity, and safety of regular polidocanol. In contrast, uniform polidocanols and their derivatives exhibit a single component, predictable physicochemical properties, and much higher bioactivity and safety than regular polidocanol.^126^

On the other hand, Movantik employs uniform PEG, which has been shown to optimize the physicochemical and pharmacokinetic properties. The uniform PEGylation of naloxegol not only improved the water solubility and bioavailability but also significantly changed the biodistribution, increasing the solubility, bioavailability, and pharmacokinetics.^127^

Moreover, it is important to point out that most of the approved PEGylated drugs include are protein-based biopharmaceuticals. There are currently only three approved PEGylated nonbiologic drugs of the small organic molecules (Naloxegol, Movantik), a synthetic peptide (Peginesatide, Omontys), and an aptamer (Pegaptanib, Macugen). However, nonbiologic drugs have also emerged as a promising target for PEGylation, as demonstrated by the candidates in the clinical development stage.^128^ However, these molecules are also subjected to PEG polydispersity, which is evident in small molecules including synthetic peptides (MW < 5 kDa), producing conjugates with different MWs, leading to difficulties in chemical characterization and purity control.^129^

Additionally, Hung et al. studied the effect of varying the number of ethylene glycol units (1–20) in A20FMDV2, which is a promising lead peptide for cancer treatment. They concluded that all PEGylated peptides displayed good stability, and the peptide with 20 ethylene units of ethylene glycol was the most stable. However, shorter PEGs were shown to be more resistant to degradation than longer PEGs. This was a first step to further understand if PEG units employed in PEGylation could be shorter than those currently employed;^130^ if future studies confirm that, it is possible could promote the use of uniform PEGs since lower uniform PEG lengths are cheaper. However, contradictory studies stated that PEGs with relatively low MWs (average 750 and 2000 Da) cross cell membranes by passive diffusion and suffer uptake more rapidly, whereas those with higher MWs (average 5000 and 20 000 Da) enter cells by passive diffusion at a low concentration and take longer to suffer uptake by cells.^122^

Moreover, other promising anticancer drug candidates such as Camptothecin (CPT) and 10-hydroxycamptothecin (HCPT), which are natural products isolated from the plant *Camptotheca acuminata*, were selectively modified with a series of uniform polyethylene glycols derivatives, including 9 ethers and 22 carbonates. These were prepared using a macrocyclic sulfate-based strategy with high efficacy, ensuring high purity and fine-tunable water solubility to the drug candidates.^131^ The same happened for drug candidate fb-PMT (NP751), a conjugate of the thyroid hormone metabolite tetraiodothyroacetic acid, which was PEGylated with uniform polyethylene glycol 36.^132^

Overall, the fact that are over 30 PEGylated drugs currently available highlights the importance and impact of PEG in drug delivery. As seen, PEGylated drugs offer several clinical advantages compared to non-PEGylated drugs, such as reduced administration frequency, improved efficacy and tolerability, and lower incidence of adverse events.^100^ However, to address the previously explained drawbacks associated with PEGylation, future attempts should be made to employ uniform PEGs in all PEGylated drugs, making it crucial to develop a more cost-effective synthetic pathway for the production of uniform PEG.

#### 5.1.2 Liposomes

Liposomes are small spherical-shaped artificial vesicles that can be created from cholesterol and natural nontoxic phospholipids. These PEG-containing vehicles for drug delivery are valid alternatives to direct PEGylation of drugs.^133^ As with proteins, PEG has been used for surface modification of liposomes and nanoparticles to increase both their stability and their in vivo circulation time.^134,135^ Therefore, PEGylation ensures that nanocarriers are not prematurely taken up by the cells, having a bigger chance of reaching and delivering the therapeutics to target diseased organs when compared to non-PEGylated ones.^136^

There are many clinically approved liposomal formulations available in the market to anticancer therapy such as Doxil/Caelyx (Doxorubicin), DaunoXome (Daunorubicin), and Marqibo (Vincristine) and a few other medications such as Ambisome (Amphotericin B) for fungal infections and DepoDur (morphine sulfate) for postoperative pain management. However, these formulations are given either intravenously and/or intramuscularly. In recent years, there has been an increased promising interest in the application of liposomes as an oral drug delivery platform due to the various advantages of an oral route.^137^ Even though the oral route is not FDA-approved to date, a lipid-based oral insulin delivery system (Oramed) that will initiate phase III clinical trials effortlessly shows the potential that lipid-based nanocarriers may have for efficient advanced oral drug delivery.^138^

PEG modifications in nanocarriers, such as liposomes, can also include the encapsulation of small interfering RNA complexes (siRNA), thus enhancing their systemic stability, increasing the half-life of siRNA in blood, and enhancing the pharmacokinetic profile. These lipids that are hydrolyzed with the pH decrease when in the endosomal environment result in the destabilization of the liposome and removal of PEG, allowing the fusion between the liposome and the endosomal membrane and releasing the siRNA in the cytosol efficiently.^139^

The first FDA-approved siRNA-based drug was Onpattro in 2018 for polyneuropathy of hereditary transthyretin-mediated amyloidosis, encouraging the interest of the pharmaceutical and academic research groups in RNA-based drugs. Furthermore, the mRNA vaccines developed by Pfizer–BioNtech and Moderna use a lipid-based nanoparticle carrier system, stabilized by a polyethylene glycol (PEG), which prevents the rapid enzymatic degradation of mRNA and facilitates in vivo delivery. Moreover, many of siRNA-based products are already in different stages of clinical trials that are expected to treat several diseases such as, for example, cancer.^140^

Furthermore, Kowalska et al. compared the release ability of liposomes composed by DSPE-PEG750 and DSPE-PEG2000 and found that longer polymer chains caused lower liposome permeability in comparison to the shorter polymer chains. The reason for this may be the fact that the PEG chains of DSPE-PEG are exposed from the liposomal surfaces and shield leakage from the liposomes. Therefore, the shielding effect of DSPE-PEG is higher for longer polymer chains.^141^

#### 5.1.3 Polymeric Nanoparticles

Polymeric nanoparticles (NPs) are particles with a size range from 1 to 1000 nm that can be loaded with active compounds entrapped within or surface adsorbed onto the polymeric core. These platforms can deliver drugs using either nanospheres or nanocapsules. Concerning the nanospheres, different forms of drug association can occur: the drug may be dissolved or dispersed within the polymeric matrix or may be adsorbed to the polymer, while nanocapsules are produced to increase the loading of lipophilic drugs, which should be entrapped by the polymeric membrane dissolved in the oily core.^142^

#### 5.1.4 Inorganic Nanocarriers

The most commonly explored inorganic nanocarriers are mesoporous silica nanoparticles (MSNs), graphene oxide (GO), black phosphorus (BP), and gold nanoparticles (GNPs). They serve as skeletons and are capable of loading and releasing drugs, keeping an intact framework in blood circulation, and holding good biocompatibility and pharmacological properties. Once again, the nanoformulation of inorganic NPs is usually conjugated with PEG to significantly decrease the clearance rates.^143^ GNPs have applications in tumor-targeting therapy by means of decorating various molecules on the surface. For example, covalent interactions (Au–S) and noncovalent interactions (electrostatic and hydrophobic interactions) are extensively employed to decorate. Furthermore, drugs or genes with thiol are linked on the surface of GNPs to apprehend drug or gene delivery; targeting groups with thiol are also decorated on the surface to enhance the targeting efficacy and GNPs can be enhanced through PEGylated to obtain long circulation.^144^ There are still many ongoing clinical tests, but there are already some promising products. The nanodrug CYT-6091, which was created by linking human TNF alpha (rhTNF) and PEG to the surface of GNPs, was tested in a phase I clinical trial on a variety of solid tumors, including colon adenocarcinoma. The results showed that the highest dose of CYT-6091 outperformed the MTD of native rhTNF by 3-fold, implying that GNPs could be promising agents in clinical application. However, numerous challenges remain in the development process, such as drug metabolism, safety concerns, in vivo efficacy, biocompatibility and stability, preparation costs, and immunogenic issues.^145^ Furthermore, there is an increasing need to pursue the synthesis of uniform GNPs that are currently being employed as contrast agents in optical imaging, photoacoustic imaging, and fluorescence imaging. Through the use of uniform GNPs, it is possible to specifically deliver agents and target tumor tissues for chemotherapy, photodynamic treatment, and other treatments to improve the efficiency of killing cancer cells, which could require avoiding disperse PEG.^87^

#### 5.1.5 Polymeric Micelles

Polymeric micelles (PMs) have been studied as drug delivery carriers for decades because they can potentially result in high drug accumulation at the target site through an enhanced permeability and retention effect. They are self-assembled microstructures formed by surfactants in an aqueous system and are usually <50 nm in diameter, composed of amphiphilic copolymers that have distinct hydrophobic and hydrophilic blocks. Usually, the most commonly used hydrophilic blocks are PEG, while the hydrophobic blocks typically are polyesters, polyethers, or polyamino acids, such as poly(l-aspartic acid) (PLA), poly(ε-caprolactone) (PCL), and poly(propylene oxide) (PPO). In aqueous solutions, the hydrophobic blocks self-associate into a semisolid core surrounded by the hydrophilic segments as a shell (corona). The hydrophilic shell provides steric stability and minimizes nonspecific uptake by the reticuloendothelial system (RES), resulting in a prolonged circulation time in the body.^146,147^ Even though PMs have been a subject of interest for drug delivery, few clinical trials have been completed or are ongoing and no products have been approved.^148^ However, important research has been conducted in this area. Shan et al. saw that the low molecular weight of PEG may contribute to the formation of compact micelles, which make them easier to be taken up by tumor cells, resulting in an enhanced antitumor effect. Therefore, micelles with low molecular weight PEG can achieve efficient delivery of drugs.^149^

#### 5.1.6 Dendrimers

Dendrimers are core–shell nanostructures with precise architecture and low polydispersity. They are synthesized in a layer-by-layer approach (expressed in “generations”) around a core unit, resulting in a high level of control over the size, branching points, and surface functionality.^150^ They are typically classified as brushed tree-like polymers having low or high molecular weights, and their structures allow them to make unique chemical conjugations. Their elevated branching points have a three-dimensional glomerular spherical shape, nanometric size, monodispersity, and lipophilicity, conferring the ability to penetrate cell walls easily and making these nanostructures ideal as a delivery system.^151^ They are able to attach to ligands, hormones, antibodies, or liposomes with loaded delivery of an active compound and have a sustained control on the liberation of these substances to specific cells. Particularly, dendrimers have been exemplified as an efficient vector for targeted neuronal disorders (such as Parkinson’s disease, Alzheimer’s disease, and epilepsy) and carcinogenic cellular gene delivery by several routes of administration, including intravenous, oral, transdermal, and ocular. The attachment of PEG to dendrimers allows the solubilization of hydrophobic drugs in the dendrimetric interiors and hydrophilic drugs in the PEG layers. Likewise, PEGylation reduces the dendrimer’s toxicity, macrophage uptake, opsonization, and agglomeration, and cosequenced improvement of the circulation time leads to their higher delivery to diseased sites.^151^ Additionally, PEGylated dendrimers have even more promising strategies because of the possibility of surface modification and hence interacting with changed functional groups.^152^ They exhibit good antiviral and antimicrobial activities due to the strong interactions that they make with a virus and bacterial membranes, and even though there are only a few available on market, several dendrimer-based drugs are under clinical trials for the treatment of cancer, bacterial infections, and even leishmaniasis for veterinary use.^153^ Uniform PEG has been employed by Zhang and co-workers by developing a synthetic approach to uniform PEGs with MWs ranging from 2 to 65 kDa (the highest MW structure of its kind ever reported). The uniform dendritic PEGs were synthesized via the PEGylation of 2,2-bis(methylol)propionic acid (bis-MPA) dendrimers and dendrons, and 11 EO units were added in each iteration. This dendritic structure facilitated degradability at pH 7.4 at 37 °C, which is an important feature for the delivery of therapeutic agents.^154^ Furthermore, highly flexible and hydrophilic groups combined with a highly branched architecture can lead to good protein resistance. To further investigate the effect of methoxy PEG molecular weight on protein resistance, PEG-acid chains with molecular weight ranging between 350 and 5000 Da were used by Benhabbour and co-workers. Protein studies comparing the original G1 dendronized surfaces to the PEG-functionalized dendronized surfaces showed that protein adsorption gradually decreased with increasing MW of PEG chains. Results also showed that increasing PEG MW beyond 2000 Da did not result in better protein resistance.^155^

#### 5.1.7 Oligonucleotide-Based Drugs

Oligonucleotide-based drugs have attracted considerable attention as promising therapeutic agents for the treatment of various human diseases; however, several issues must be overcome in the development of oligonucleotide-based drugs. For example, unmodified single-stranded DNA and single-stranded RNA are quite unstable, especially in vivo. Double-stranded RNA, such as small interfering RNA (siRNA), in contrast, is stable under cell culture media, which contains a low concentration of serum. Rapid renal clearance presents another problem since short oligonucleotides fall from the kidneys because their molecular weights are far less than the molecular weight threshold in renal filtration, which is about 65 kDa. Furthermore, there is a limited tolerance against enzymatic degradation.^156^ Therefore, PEGylation of these drugs resulted in enhanced gene silencing for an siRNA which had only average efficiency in its wild-type form. For siRNA, PEG chains with molecular weights between 2 and 20 kDa have been attached to the oligonucleotides. However, these polymer sizes are usually disperse, which yields mixtures of conjugates with different PEG chain lengths, complicating purification and analyses. To avoid those problems, siRNA has been conjugated with short PEG chains with defined length and size.^157,158^ Moreover, antisense oligonucleotides can be modified with short PEG12 chains at the 3′- and 5′-end without any loss in gene silencing activity.^157^

#### 3.1.8 PEGylated Adenovirus-Based Vaccines

Adenoviruses have been used as vehicles for recombinant DNA-based vaccines due to their ability to stimulate strong innate and adaptive immune responses. However, use of the most common adenovirus serotype 5 is limited due to a significant portion of the population having pre-existing immunity to it. Nonhuman serotypes, such as chimpanzee adenovirus C7, or rare human serotypes, such as serotype 35 or 11, have been used but have their own safety and production issues. PEGylation of adenovirus vectors has been shown to efficiently induce transgene expression even in the presence of pre-existing immunity; therefore, the covalent attachment of many different forms of PEG can significantly attenuate the immune response against the virus capsid and improve transduction efficiency in some tissues alone.^159^

#### 5.1.9 Antibody–Drug Conjugates (ADCs)

Antibody–drug conjugates (ADCs) are a novel approach to cancer treatment that combines the selectivity of monoclonal antibodies (mAbs) with the cytotoxic properties of powerful anticancer drugs. This emerging therapeutic strategy holds great promise for the treatment of cancer by enabling the delivery of potent drugs directly to cancer cells while minimizing the risk of systemic toxicity.^160^ However, ADCs have shown solubility issues that lead to aggregation and drug loading limitations and impact the pharmacokinetics and pharmacodynamics (PK/PD). Considering the significant achievements of PEGylation in protein therapeutics, the use of uniform PEG was employed to address this issue of low solubility, helping to overcome the hydrophobicity challenges.^161^ Even though this is an emerging field still in need of more research, some achievements regarding the use of PEG have already been obtained. Smaller PEG chains (≤PEG7) have showed great promise, suggesting that there is a tight correlation between the polymer length and its placement in the linker structure. A PEG chain with a more flexible design would be more effective in masking the hydrophobicity of the drug payload compared to a rigid and elongated structure that separates the drug from the antibody.^162^

## 6 Immunology

Despite the FDA’s approval of several PEGylated products, some publications suggest that the PEG component in these products could be immunogenic and lead to enhanced blood clearance (ABC), thereby reducing their efficacy.^12,13,163^ This is due to the report of some PEGylated therapeutics inducing the formation of anti-PEG antibodies (APA) in patients who have never received PEGylated drugs but have consumed products containing PEG. Such an occurrence could result in decreased effectiveness and an increase in adverse events.^164,165^

APA, IgM, and IgG can neutralize the therapeutic effect of the drug, leading to a reduction in clinical efficacy. Additionally, they may cause adverse immune effects, such as the acceleration of blood clearance of the PEGylated drug (ABC phenomenon), resulting in a loss of efficacy, and hypersensitivity reactions that could potentially lead to anaphylactic shock and, in severe cases, even death.^166^ Yokoyama and colleagues discovered that induced IgM recognizes the interface between the PEG chain and the hydrophobic chain rather than the PEG main chain itself. However, this finding only applied to polymeric micelles.^167^ Roffler et al. investigated how anti-PEG antibodies cause hypersensitivity reactions. They found that anti-PEG IgG but not anti-PEG IgM antibodies can induce symptoms of hypersensitivity in mice, such as hypothermia, reduced systolic, and diastolic blood pressure, and altered lung function. IgG antibodies can trigger hypersensitivity reactions by binding to PEG on PEGylated nanoparticles, liposomes, or macromolecules and interacting with FcγRs on basophils, neutrophils, and macrophages, which leads to the release of platelet-activating factor and histamine. Additionally, they speculate that immunoglobulins in the protein corona on the surface of nanoparticles may contribute to this hypersensitivity reaction through a similar mechanism.^168^

To address this concern, the FDA released guidelines in 2014 pertaining to the evaluation of immunogenicity for therapeutic protein products. These guidelines emphasize the significance of testing for two categories of antibodies: those that target the therapeutic protein and those that target PEG. Consequently, it is now a standard practice in the research and development of PEGylated products to employ assays that can measure both the quantity and the potency of anti-PEG antibodies subsequent to the administration of a PEGylated product.^169^

The way in which APA specifically binds PEG remains poorly understood. Huckaby et al. delved into the structural characteristics of an antibody (APA) when it forms a complex with its polymer antigen, which promoted more understanding of the mechanism of action against PEGylated therapeutics. The APA forms a dynamic ring structure, enabling it to capture PEG and further stabilizing the binding by wrapping PEG around the Trp96 residue of the heavy chain. This unique binding mechanism also explains cross-reactivity with other C–C–O backbone polymers.^170^ Later, Nguyen et al. presented the mechanism for specific binding of antibodies to mPEG. The binding is achieved through van der Waals and H-bond interactions with aromatic amino acids, providing methoxy specificity to the antibody. They found an unique methoxy specificity of the antibody h15-2b. These insights are valuable for understanding how antibodies interact with PEG and can aid in the development of high-affinity anti-mPEG antibodies for various applications, including targeted mPEG-nanomedicine.^171^

The process of ABC begins with the proliferation and differentiation of specific B cells in the marginal zone of the spleen in T-cell-independent manner, resulting in anti-PEG IgM formation with a first contact with PEG. This will result in a complement activation and clearance from systemic circulation when the second injection is administered.^13,172^ This phenomenon can also be observed in healthy individuals that did not receive PEG-containing drugs due to the frequent use of PEG-containing or PEG-coupled products that are common ingredients in personal care, beauty, and household cleaning products (e.g., soap, sunblock, cosmetics) as well as processed foods.^173^

In order to see if the route of administration of drugs can impact how the ABC phenomenon occurs, Zhao et al. demonstrated that the induction of the ABC phenomenon was limited to not only intravenous injections but also when two consecutive doses of PEGylated drugs are administered in different approaches. For example, rats given subcutaneous PEGylated solid lipid nanoparticles followed by intravenous injection caused rapid clearance of the doses of the PEGylated drug, proving that this effect is independent from the root of administration.^174^

In order to understand if the immune reactions could be decreased, Shimizu et al. showed that using hydroxyl PEG instead of methoxy PEG in liposomes would decrease the chances of an immune reaction after one injection. However, when these liposomes are given multiple times, they still face a significant issue known as the ABC phenomenon. These findings emphasize the importance of thoroughly testing complement activation and monitoring antipolymer antibody production before using polymer-modified treatments in clinical settings.^175^ Also in accordance, Sherman et al. concluded that the accelerated clearance and the consequent loss of efficacy of mPEG conjugates of therapeutic agents might be decreased by synthesizing next-generation versions of these drugs with monofunctionally activated derivatives of hydroxy-PEG instead of mPEG. The potential advantages of HO-PEG conjugates may be justified by the decreased risk of treatment-limiting immune responses.^176^ Later, Sherman et al. proved that the clinical use of hydroxyl-PEG–protein conjugates could induce less intense anti-PEG immune responses than the use of mPEG–protein conjugate.^177^ Wan et al. studied whether branching PEG would suppress or decrease the anti-PEG immune responses; however, they concluded that branching PEG has an insignificant effect on PEG immunogenicity.^178^ Moreover, different PEG chains length were studied to further extend how they can affect anti-PEG Ab induction. The minimum MW of an anti-PEG epitope on PEG was reported to be 750 Da. On the other hand, PEG with lengths of 30, 20, and 5 kDa induced a comparable higher anti-PEG IgM response.^179^ Recently, Lin et al. published that anti-PEG antibody clearance of PEGylated compounds depends on the PEGylation architecture. By this means, compounds with multiple mPEG chains are easily cleared, like enzymes and nanomedicines that are modified with many mPEG molecules.^180^ McSweeney et al. developed an approach where high MW PEGs were administered in animals, which caused a safe and effective prolonged circulation and efficacy of the PEGylated medicines. They found that the infusion with high MW PEG was a practical strategy to control APA-specific B cells, showing the possibility of using this approach as a repeated intervention, possibly prior to each dose on a PEGylated drug in patients with high APA titer.^181^ A different approach was made by Hsieh et al., who studied the preinjection of anti-PEG monoclonal antibodies before administration of PEGylated liposome, nanoparticles, or proteins in mice. This preimmunization of mice with a PEGylated protein to generate polyclonal anti-PEG antibodies caused similar accelerated blood clearance, indicating that determining the APA before taking the PEGylated drug is very important.^182^ Later, in 2021, the same authors saw that free PEG has the ability to prevent induction of APA by PEG-liposomes over many weeks, indicating that it would also be effective if administered 14 days in advance of PEG-liposome dosing.^183^ Other strategies already in clinical trials were developed as rapamycin-loaded PLGA particles that were coadministered with a PEGylated protein, reducing the APA effect. This is currently in phase II clinical trials, and although this therapy is well tolerated, a small number of patients still showed response in the therapy.^184^

Moreover, Xu et al. prepared cleavable PEG-cholesterol derivatives by conjugating PEG to cholesteryl hemisuccinate (CHEMS) and to cholesteryl chloroformate (CHM) with a single ester bond. They hypothesize that the presence of physiological esterase in rats cleaved the linkage of the PEG to lipids and gradually removed this copolymer from the surface of nanocarriers, thus forming exposed nanoparticles. Multiple administrations of liposomes coated on the surface with CHEMS- and/or CHM-PEGs decreased the induction of the ABC phenomenon in the same rat models.^185^ On the other hand, Pun and Roffler et al. found that the substitution of the l-amino acid peptide for its d-amino acid enantiomer significantly attenuated the anti-PEG antibody generation and toxicity, permitting repeat injections.^186^

Another remaining issue is that there is a need for a standardized, sensitive, and quantitative method of APA detection. Early techniques were limited, and while enzyme-linked immunosorbent assay (ELISA) is now the preferred method, varied protocols and surfactant use have hindered standardization.^187^ Animal experiments should induce APA with therapeutic doses of PEGylated substances to reflect clinical relevance and understand their influence on accelerated drug clearance and pseudoallergic reactions. A standardized ELISA could screen patients for αPEG Abs before PEGylated treatments, aiding in personalized medicine. But, the scientific community must address knowledge gaps and misconceptions about APA given PEG’s growing use in consumer products and therapies.^188^

Sellaturay et al. pointed out that patients with PEG allergy should identify the PEG MWs to be avoided and list the medications that provoked allergy reaction, for example, if a patient reacted to PEG 20000 but tolerated medications containing PEG 6000; therefore, it only has high risk of anaphylaxis when the molecular weight is above 6000.^189^

Despite the numerous benefits that PEG offers in drug delivery, its immunogenicity has emerged as a significant concern. Ongoing studies are dedicated to tackling this issue, underscoring the importance of PEG as a vital polymer in drug delivery. However, additional research and development are imperative to surmount this challenge and enhance the effectiveness of PEG in clinical applications.

## 7 Alternatives to PEG

### 7.1 Natural Source Polymers

The challenges mentioned above raised a need to search for alternative polymers for drug delivery. Natural source polymers as heparin, polysarcosine, dextran, polyamino acids, and chitosan have been studied through the years. They have comparable shielding effects to PEG without inducing immune response. However, they still lack more studies as drug delivery vehicles.^190^ Heparin (HEP) is a carbohydrate-based polymer that presents no immunogenicity and shortens the drug’s half-life in the bloodstream. The use of HEP has been proven to provide shielding properties in coating nanoparticles; however, its clinical setting is not applied since it is still poorly studied.^190,191^ Polysarcosine is a biobased, nonionic, and hydrophilic polypeptoid that has low toxicity and an excellent stealth effect. Moreover, it has been tested for efficient mRNA delivery, able to successfully substitute PEG-lipids in lipid formulations.^192^ Chitosan is a mucopolysaccharide derived from chitin, being produced by the deacetylation of chitin. The degree of deacetylation affects its hydrophobicity, solubility, and toxicity since a higher degree of deacetylation shows toxicity. Moreover, low molecular weights are less toxic and appear to have a lower degradation rate. On the other hand, high molecular weights appear to be toxic and less soluble. This polymer has been tested in drug delivery, enhancing the therapeutic efficacy of drugs. It was seen that it is a biocompatible and biodegradable polymer with mucoadhesive properties and absorption-enhancing capability, making this polymer a great substitute of PEG. However, the toxicity and safety issues of chitosan NPs and their manufacturing techniques needs to be investigated very closely.^193^ Dextran is a nonmammalian polysaccharide being obtained by expression in bacteria. It is highly water soluble and easily functionalized though its reactive hydroxyl end groups. It has been tested for drug delivery purposes; however, it lacks dispersity control since the average molecular weight distributions can vary depending on the conditions and strain of bacteria used for expression.^194^ Polyamino acids are biodegradable polymers with prolonged blood circulation time. Examples of polyamino acids include poly(glutamic acid) (PGA), which is already in clinical trials as PGA-paclitaxel conjugate. PGA has similar pharmacokinetics and biodistribution profiles as PEG, presenting no toxic effects with improved stealth properties.^195,196^ Other examples of attractive alternatives to PEG include poly(hydroxyethyl-l-asparagine) (PHEA) and poly(hydroxyethyl-l-glutamine) (PHEG), which have already been applied in drug delivery systems. Coating of liposomes with PHEG and/or PHEA extended the circulation half-life to a similar extent as PEG.^197^ This class of polymers is degraded in vivo to their corresponding amino that can be metabolized by physiological pathways. However, the major downside is complemented activation; nevertheless, this effect may be tolerable in clinical trials since it apparently leads to only moderate hypersensitivity reactions.^10^

### 7.2 Zwitterionic Polymers

Zwitterionic polymers, such as poly(carboxybetaine) (PCB) and poly(sulfobetaine) (PSB), have been proposed as PEG alternatives since they present high hydration, high resistance to nonspecific protein fouling, and low immunogenicity. These polymers usually have both cationic and anionic groups in the structures and have revealed efficient antiprotein absorption and appealing biological effects. They have been tested in coating gold nanoparticles and proteins being more stable in the bloodstream with a longer circulation time than PEG–NPs. These classes of polymers have tunable properties for various purposes in biomedical applications; however, they still lack immunology studies.^198,199^ They are synthesized by copolymerization of cationic and anionic monomers or by polymerization of zwitterionic monomers. Besides, they can also be obtained via postpolymerization modification, such as click reaction between pendent groups and zwitterionic molecules. In the case of PCB, the polymer is synthesized by repeated CB-based monomers, which has a cationic trimethylammonium group and an anionic carboxylic group in each unit, resulting in abundant carboxylic groups for drugs or targeting ligand conjugation. Like PCB, PSB has a pair of cationic trimethylammonium groups and anionic sulfonate groups in each unit; however, this polymer is considered temperature responsive and possesses an upper critical solution temperature due to the self-associations among the charged groups.^200,201^

### 7.3 Polyoxazolines

Polyoxazolines like poly(2-oxazoline)s (POX), poly(2-methyl-2-oxazoline) (PMeOx) and poly(2-ethyl-2-oxazoline) (PEtOx) are hydrophilic polymers studied for half a century and their synthesis is based in cationic ring-opening polymerization with PDI < 1.2. Although these polymers have better renal clearance, their synthesis acquires high cost and still lack FDA approval.^198,202^

### 7.4 Vinyl-Based Polymers

A vinyl-based polymer such as poly(*N*-vinylpyrrolidone) (PVP) is a nonionic, biocompatible, and hydrophilic polymer with lower degradation than PEG, capable of suppressing immune activation and is already widely used in pharmaceutical and food industries. The PVPs that are already commercially available have a MW average between 400 and 100 kDa, and multiple types of inorganic NPs can be synthesized and stored for months without aggregation by addition of PVP during the preparation. Nonetheless, these PVP polymers are inert without functional groups in the end of polymer chains and are synthesized using radical polymerization with broad polydispersity, having uncontrolled chain-end functionalities, making them unsuitable for polymer therapeutics.^200,202^ Poly(vinyl alcohol) is a water-soluble polymer widely used as a component of biomaterials and drug delivery systems. It exhibits surface-active properties, however, and cannot be synthesized by direct polymerization of vinyl alcohol because of the unstable nature of this monomer. Therefore, it is usually synthesized by polymerization of poly(vinyl acetate), containing residual vinyl acetate groups that impact its physicochemical properties.^203^

### 7.5 Polyacrylamides

Polyacrylamide-based polymers such as poly(*N*-(2-hydroxypropyl)methacrylamide) (PHPMA) and poly(*N*,*N*-dimethyl acrylamide) (PDMA) have also been used to replace PEG. PHPMA is a nonionic, biocompatible, and water-soluble polymer with defined applications in drug delivery being used in drug and protein conjugation, self-assembled nanoparticles, and hydrogels. PHPMA has several advantages over PEG since it does not show dependent immune responses, rapid clearance after repeated injections, and potential oxidation. A disadvantage is that its synthesis relies on conventional free radical polymerization, atom transfer polymerization (ATRP), and reversible addition–fragmentation chain transfer (RAFT). PHPMA has demonstrated an outstanding preclinical efficacy as a carrier for chemotherapeutic drugs and has recently entered clinical trials, possibly one of the best candidates for PEG alternatives.^204,205^ Poly(2-hydroxyethyl methacrylate) (PHEMA) has excellent biocompatibility with a proven nonirritation potential for mucosal tissues (application in contact lens industry). A disadvantage is that PHEMA is relatively hydrophobic and is not soluble in water, which can obstruct diffusion of PHEMA-decorated nanoparticles through mucus.^203^

### 7.6 Polyglycerols

Polyglycerols (PGs) have the advantage of providing hyperbranched structures with high degrees of modification. Since these are being made available to broader industries, there was a concern about the risk of using polymers that are also formulated into everyday products, thus leading to generation of acquired immunity against them. However, after studies, PGs have been seen to improve the circulation time and do not induce enhanced blood clearance ABC after repeated exposure. A drawback is that these polymers are not biodegradable and present high accumulation in tissues.^10,190,198^

### 7.7 Conclusions and Future Prospects

Overall, several alternative polymers to PEG have been developed and have shown promise in drug delivery applications with ongoing studies to determine if they can replace PEG as the gold standard polymer in drug delivery.

## 8 Stability

In drug delivery, it is crucial to control the stability of the polymers, both in treatment and in storage. Therefore, in the past years PEG degradation has been studied to further understand whether chemical changes could occur when induced by oxygen, water, heat, radiation, or even mechanical forces.

These studies started in the 1960s, where it was found that oxygen would promote the oxidation of PEG, forming as side products H_2_O, CO_2_, CH_2_O, CH_3_CHO, and HCOOCH_3_. Moreover, a thermal-oxidative degradation promoted a chain scission of PEG due to the same hydroperoxides, resulting in degradation of the polymer chain that can occur with PEG in the solid state and in solution.^206,207^ Later, in 1996, Han et al. studied this same type of degradation and discovered that the thermal degradation products could be suppressed using 2,2″-methylene-bis(4-methyl-6-*tert*-butylphenol) (MBMTBP) as an antioxidant.^208^ Later, in 2006, vacuum drying of PEG solutions at low pressures (0.1 mmHg) was employed as an effective method to purify PEGs, significantly improving the chemical stability of the active ingredients.^209^ Moreover, PEG can also degrade under photoinduced mechanisms. The peroxy radicals react intermolecularly, and dissociation of hydroperoxides happens very fast, producing formates. Simultaneously, the peroxy termination reaction rate constant increases as polymer degradation continues.^210^

Morlat et al. compared both thermal and photo-oxidation degradation pathways and analyzed which are the expected side products involved. They concluded that hydroperoxide formation is prone to happen by the thermal oxidation of PEG since the hydroxyl radicals can be formed by decomposition of the hydroperoxides, reacting with macroalkoxy radicals, producing esters. This will damage the polymer chain since the carbonylated products are formate end groups that lead to the formation of carboxylic acids, which will influence the PEG molecular weight. On the other hand, peroxide radicals are more prone to occur in the photo-oxidation degradation. The decomposition of hydroperoxide leads to the formation of alkoxy radicals that can cut the chain.^211^

Payne et al. analyzed these degradation pathways in uniform and disperse PEGs to understand if the polydispersity would contribute to the degradation of the polymer. They found that there was a consistent oxidative behavior for both PEGs; however, assessment of the degraded products was more easily found in uniform PEG since they are easier to analyze. Therefore, they were able to analyze the degradation products that occur through oxidative degradation, as seen in Table 4.^117^

**Table 4:**
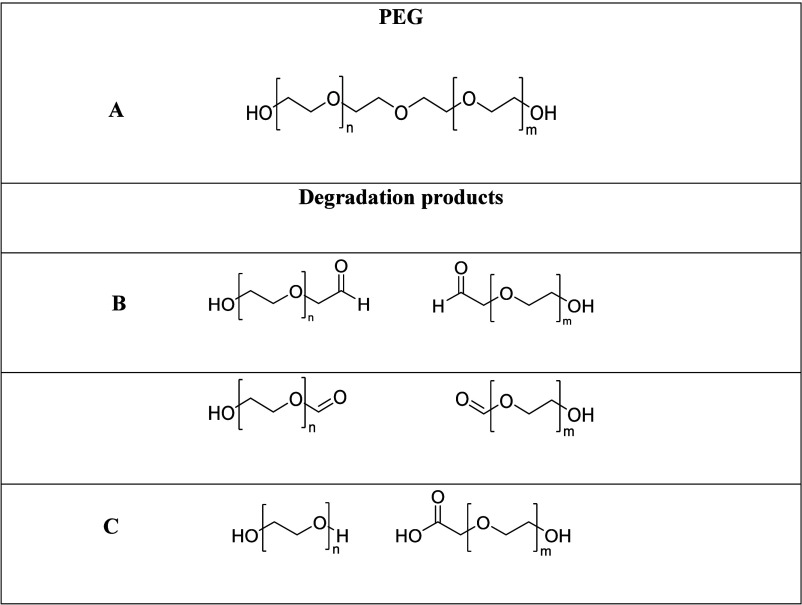
Degradation Products of PEG Oxidation^a^

a (A) Diol oxidation results in the formation of an aldehyde. (B) Polymer chain scission can yield a monoformate PEG and a hydroxymethyl group (hemiacetal) that quickly breaks down to form an alcohol (dihydroxy PEG) and formaldehyde. (C) Carboxylic acid end group formation.

## 9 Challenges and Future Perspectives

Polyethylene glycol (PEG) has become a gold standard polymer in various biomedical applications due to its biocompatibility, hydrophilicity, and versatility. However, despite its widespread use, PEG has several challenges that need to be addressed to optimize its performance in drug delivery and other biomedical applications. In this section, we will discuss some of the key challenges associated with PEG and the ongoing efforts to address them.

One of the major challenges associated with PEG is its immunogenicity. PEG is a foreign molecule to the human body, and repeated exposure to PEG can lead to the formation of anti-PEG antibodies. There are many key factors that affect anti-PEG immune response and the ABC phenomenon that have been reported and explored, including not only the MW but also the dosage method and preparation of PEGylated agents and the chemical nature of the terminal end group. For example, among the frequently used PEG end groups, the binding affinities of formed Abs increase in the following order: hydroxy (−OH) < amino (−NH_3_^+^) < methoxy(−O–CH_3_) < butoxy (−O–(CH_2_)_3_)–CH_3_) < *tert*-butoxy (−O–(CH_3_)_3_).^9^ To corroborate, other research has shown that hydroxyl PEG-modified liposomes (PL-OH) efficiently attenuated the anti-PEG IgM response in vitro, making PL less recognizable by anti-PEG IgM compared with other PLs. These findings raised the possibility that PL-OH could attenuate the induction of the ABC phenomenon.^122^

Therefore, through the years, research in the presence of anti-PEG antibodies has raised awareness that PEGylated products face a significant challenge for clinical approval. One specific example is are allergic reactions of the COVID-19 RNA vaccines developed by Pfizer and Moderna which are believed to be correlated to PEG-containing lipid formulations.^135^ Further investigations have revealed that the hypersensitivity reactions caused by PEG and its derivatives in the vaccine formulation may be a significant cause of the adverse side effects.^212,213^ Furthermore, employing high molecular weight PEG is not advised since it can enhance a secondary consequence of immunogenicity, and the PEG chain length affects the extent of anti-PEG Ab induction and promotes substantial tissue accumulation.^11^ On the other hand, low MW PEG can have a positive relationship between the length of PEG and anti-PEG Ab induction, while PEGs with MWs of 20 000 and 30 000 show higher anti-PEG response; however, the oxidative side products of lower molecular weight oligomers have been shown to have toxic side effects.^214^

Furthermore, PEGylated products have been in the market for the last 20 years as a critical ingredient in daily products, medicine, and surfactants as well as in industrial and clinical applications.^10^ This wide usage has shown that around 70% of the general population possesses pre-existing APA with detectable levels of anti-PEG IgM or IgG, emphasizing the potential for rapid immune stimulation upon treatment with certain PEGylated therapeutics.^181^

Another challenge associated with PEG is its polydispersity. Commercially available PEG is a mixture of PEG molecules with different molecular weights and chain lengths, resulting in a disperse distribution. This can lead to variations in the pharmacokinetics and biodistribution of PEGylated drugs, reducing their efficacy and potentially increasing their toxicity.^10^ To overcome this challenge, researchers are exploring various methods to synthesize uniform PEG with well-defined chain lengths and molecular weights; however, these synthetic pathways are still based on iterative steps with purifications in between that promote another challenge, which is the high price of uniform PEG. Therefore, it is necessary to develop a more cost-effective pathway to synthesize this polymer that could eventually attenuate the immunologic side effects.

Furthermore, due to the inherent features of PEG, there are a number of concerns with PEGylation. These include issues with bioactivity loss, the heterogeneity of the conjugation sites and degrees, the consequent difficulty in separation, and the low recovery rate of the PEGylated protein. In an effort to preserve the biological activity, different modification chemistries, varied PEG structures, and derivatives have been studied with incoming improvements.

Nonetheless, even though PEG is a highly versatile and widely used polymer in biomedical applications, it has several challenges that need to be addressed to optimize its performance. Ongoing research efforts are aimed at addressing these challenges and developing more effective and cost-efficient methods for the synthesis of uniform PEG.

## 10 Conclusion

This review provides an overview of different strategies for preparing uniform PEGs while highlighting their limitations.

Through the development of new synthetic approaches, it has become possible to produce PEGs with controlled molecular weights and narrow polydispersity, which have shown great potential in a variety of biomedical applications. These uniform PEGs have demonstrated improved solubility, biocompatibility, and reduced immunogenicity compared to their disperse counterparts. Additionally, the ability to introduce functional groups or modifications to the PEG chain has allowed for greater versatility in their use as drug delivery vehicles, imaging agents, and more. However, despite its success, there are still challenges to overcome in terms of scalability of these synthetic methods since almost all of the developed processes included scales below 100 g, with the exception of that by Zhang et al., who developed a synthetic pathway yielding PEGs over a 100 g scale.^17^ This approach was also the one that produced higher length PEGs (64 EO units) without any significant loss of yield, showing to be the most promising. On the other hand, most of the remaining developed strategies have several drawbacks including reduced yields when dealing with higher molecular length PEGs (over 20 EO units) and unsuccessful isolation procedures having the need for chromatographic isolation. Therefore, despite the advancements in PEG synthesis, several challenges remain to be addressed. This highlights one of the major hurdles which is the cost associated with PEG production. Many developed synthetic pathways involve multistep processes, contributing to higher production expenses, raising the price disparity between disperse and uniform PEGs. For instance, the cost of disperse PEG2000 is approximately 0.05€ per gram,^215^ whereas uniform PEG2000 can be as high as 950€.^216^ Closing this price gap and making uniform PEGs more economically accessible is a crucial objective for further advancements in the drug delivery field.

Moreover, while PEGs are widely used and considered safe in many applications, assessing the balance between their benefits and potential drawbacks is crucial. A big concern regarding using PEGs in cosmetics and drug delivery is the potential accumulation in the body. PEGs are primarily excreted through the kidneys, but evidence suggests that prolonged exposure to high levels of PEGs may result in their accumulation in specific tissues. Nonetheless, extensive studies and regulatory assessments indicate that PEGs do not pose significant health risks when used in appropriate concentrations.

Moreover, differentiating between facts and misconceptions regarding PEG safety is essential. Despite concerns about their potential carcinogenicity, toxicity to the liver and kidneys, and allergic characteristics, scientific evidence and regulatory evaluations affirm the general safety of PEGs when used as intended. However, ongoing research is needed to comprehend further the potential long-term effects and accumulation of PEGs in the body, ensuring their safe utilization in diverse applications. Furthermore, it is important to remain cautious and consider individual sensitivities or allergic reactions to PEG-containing products since the immunogenicity of PEG has led to unwanted immune responses and treatment failure. Therefore, it is necessary to explore alternative strategies that can provide the benefits of PEGylation without triggering immunological reactions. To this end, alternative approaches such as zwitterionic polymers, polyacrylamides, polyglycerols, and vinyl-based polymers have been developed and show promising results. Nevertheless, these alternatives still exhibit certain limitations that prevent them from matching the desirable properties offered by PEG.

The environmental impact of PEGs is a significant concern, primarily due to their slow environmental degradation. Although PEGs are not inherently toxic to the environment, their production processes may involve using raw materials that can be harmful. Therefore, it is crucial to focus on improving the sustainability of PEG production by adopting greener manufacturing practices that reduce hazardous chemicals, minimize energy consumption, and limit waste generation. Continually exploring innovative solutions and responsible practices will help minimize the environmental footprint of PEGs while retaining their valuable properties for diverse applications.

In conclusion, the significance of uniform PEG cannot be overstated because of the clear advantages that its precise molecular weight and uniformity provide in a variety of applications. With continued advancements in improved synthetic pathways and the development of more cost-effective production methods, the potential to enhance drug delivery and other applications of uniform PEGs becomes increasingly promising.
